# Gender differences in the association between oral health literacy and oral health-related quality of life in older adults

**DOI:** 10.1186/s12903-022-02237-8

**Published:** 2022-05-25

**Authors:** Chia-Jung Lee, Mu-Hsing Ho, Jee Young Joo, Jed Montayre, Yen-Kuang Lin, Chia-Chi Chang, Megan F. Liu

**Affiliations:** 1 School of Nursing, College of Nursing, Taipei Medical University, Taipei, Taiwan; 2 School of Nursing, Li Ka Shing Faculty of Medicine, The University of Hong Kong, Pok Fu Lam, Hong Kong; 3 College of Nursing, Gachon University, Incheon, Korea; 4 School of Nursing and Midwifry, Western Sydney University, Campbelltown, NSW Australia; 5 Graduate Institute of Athletics and Coaching Science, National Taiwan Sport University, Taoyuan, Taiwan; 6 School of Gerontology Health Management, College of Nursing, Taipei Medical University, 250 Wuxing St., Taipei, 11031 Taiwan; 7 College of Nursing, and College of Interdisciplinary Studies, Taipei Medical University, Taipei, Taiwan

**Keywords:** Gender, Older adults, Oral health, Oral health literacy, Quality of life

## Abstract

**Background:**

Poor oral health affects quality of life; oral health literacy studies are increasing as it plays an essential role in promoting oral health. However, little is known regarding the gender differences in oral health literacy and oral health-related quality of life (OHRQoL) among older adults. This study aimed to explore the gender differences in oral health literacy and OHRQoL among community-dwelling older adults in Taiwan.

**Methods:**

A cross-sectional study design with convenience sampling was undertaken to recruit participants at two community service centres. Data were collected using a structured survey consisted of the demographic characteristics, instrumental activities of daily living, nutrition assessment, oral health literacy and OHRQoL. The logistic regression was used to examine the gender differences in the relationship between oral health literacy and OHRQoL.

**Results:**

A total of 202 participants completed the survey. Of which 56.4% (n = 114) were female. Logistic regression analyses showed that after controlling for age, instrumental activities of daily living, nutrition, education level, and average monthly income, better oral health literacy was associated with better oral health quality of life (*p* = 0.006) in men.

**Conclusions:**

The relationship between oral health literacy and OHRQoL was only significant for men. No significant relationship between women’s oral health literacy and their OHRQoL. However, good OHRQoL is an integral part of overall health, but it is affected by differences in oral health and the accessibility of healthcare services. We suggest that gender-specific oral health literacy education should be offered through community health-education programs.

**Supplementary Information:**

The online version contains supplementary material available at 10.1186/s12903-022-02237-8.

## Background

With advances in medical technology, population aging has become a trend affecting countries all over the world, and accordingly, the health of older adults has become an important area of concern. The World Health Organization (WHO) points out that oral health is a key indicator of overall health, quality of life, and wellbeing. As a result, several countries have formulated measures for improving oral health. As the US Department of Health and Human Services pointed out in Healthy People 2030 [1], good oral health improves a person’s ability to speak, smile, smell, taste, touch, chew, swallow, and make facial expressions to show their feelings and emotions. In 2015, Taiwan’s Department of Mental and Oral Health in the Ministry of Health and Welfare announced its National Oral Health Promotion Plan (2017–2021), which indicates that oral health has also become an essential part of health-promotion policy in Taiwan.

Previous studies have shown that oral health literacy is related to maintaining good oral health [2, 3]. Lower health literacy is associated with poorer patient outcomes. People with lower health literacy tend to exhibit poorer utilization of preventive screening, poorer compliance with doctors’ orders, less appropriate use of drugs, higher medical expenses, higher hospitalization rates, greater demand for emergency services, and higher risk of death [4, 5]. The US Department of Health and Human Services defined oral health literacy as “the degree to which individuals have the capacity to obtain, process and understand basic oral health information and services needed to make appropriate health decisions [6].” This definition is consistent with the concept of general health literacy.

According to the WHO, oral health is a key indicator of quality of life, and oral health-related quality of life (OHRQoL) refers to an individual’s perception of their oral health [7]. Studies have also shown that oral health is associated with OHRQoL and OHRQoL is considered as a subjective oral health outcome [8–10]. Oral health literacy and OHRQoL are affected by demographic factors such as age [11–13], education level [12, 14–16], monthly income [17, 18], nutrition [19–21], and instrumental activities of daily living [8]. However, the effect of gender on oral health literacy and OHRQoL has not yet been determined.

The 2011–2016 Oral Health Surveillance Report published by the Centers for Disease Control and Prevention (CDC) in 2019 showed that 17.7% of men and 16.9% of women over 65 years old were completely toothless. According to the report on the 2015–2016 Oral Health Survey of Adults and the Elderly released by Taiwan’s Department of Mental and Oral Health, Ministry of Health and Welfare, 5.1% of men and 3.7% of women aged 65–74, and 9.6% of men and 10.2% of women aged 75 or older, were toothless. Some studies have documented significant gender differences in oral health literacy [22, 23], but others did not find any gender differences [24–28]. The relationship between gender and OHRQoL has not yet been fully established. Some studies have reported differences in gender among oral health conditions [29–31], but have found no significant differences between the gender and oral health literacy [32, 33].

Oral health and OHRQoL can be affected by oral health literacy. However, studies evaluating these factors or the association between them in older community-dwelling adults in Taiwan are limited. Therefore, the purpose of this study was to identify the gender differences in the relationship between oral health literacy and OHRQoL among community-dwelling older adults in Taiwan.

## Methods

### Aim and study design

This study aimed to explore the gender differences in oral health literacy and OHRQoL among community-dwelling older adults in Taiwan. A cross-sectional study design with convenience sampling was undertaken to recruit participants at two community service centres in Taipei City, Taiwan.

### Participants

Eligible participants were invited to participate in this study according to the selection criteria. The inclusion criteria were the ability to communicate in Chinese or Taiwanese, full conscious awareness, the ability to read the questionnaire unaided, agreement to participate in the research, and an age of 65 years or older. The exclusion criteria were illiteracy and an inability to communicate normally due to hearing impairment.

To ensure that the rights and welfare of the participants were protected and ethical guidelines were followed, the investigator first obtained oral consent from the participants before explaining the study procedures to them. After the investigator had verified the procedure with the participants and received their informed consent, the participants completed the questionnaire. The study protocol was reviewed and approved by the Joint Institutional Review Board of the University (approval number: N202012055).

The sample size was estimated using G Power 3.1.9.7 software given the following parameters: Cohen’s *d* (effect size) = 0.15, *α* (error probability) = 0.05, and 1 − *β* (power) = 0.95 [34]. Six independent variables namely oral health literacy, age, education, income, nutrition, and instrumental activities of daily living were entered to estimate the sample size of 146 was required. In a previous study related to OHRQoL, the questionnaire response rate was found to be 71.62% [8]. Based on an attrition rate of 30%, the estimated number of participants that needed to recruit was 200.

### Data collection

A structured survey was used to conduct data collection, which incorporated scales translated by Taiwanese scholars and questionnaires developed by Taiwanese researchers. The research instrument included demographic data, an oral health literacy scale, an oral health impact profile, nutritional screening, and instrumental activities of daily living.

#### Demographic characteristics

The demographic data collected were gender, age, education level and individual monthly income.

#### Oral health literacy

We used the Mandarin version of the Oral Health Literacy Adult Questionnaire (OHL-AQ), which was translated by Ho et al. (2020) [35] from the original version, which was developed by Naghibi Sistani et al. (2014) [36]. The expert content validity index (CVI) for all items was > 0.90. This questionnaire consisted of 17 questions. With one point assigned to each question, the minimum and maximum possible scores were 0 and 17, respectively. A score of 9 points or below is considered to indicate insufficient oral health literacy, while scores of 10–11 points and 12–17 points indicate moderate and sufficient oral health literacy, respectively. The CVI of this scale was 0.95, and Cronbach’s alpha was 0.78. According to a study conducted in Taiwan (Ho et al., 2019), the mean oral health literacy scores for community-dwelling older adults (65–80 years old) and middle-aged people (45–64 years old) were 9.77 ± 3.35 and 12.20 ± 3.10, respectively. In the current study, the focus was on community-dwelling older adults who had a typical level of oral health literacy, so participants who scored 12–17 and were thus considered to have good oral health knowledge were excluded from further analyses.

#### Nutrition

We used the Mini Nutritional Assessment—Short Form (MNA-SF) for nutritional screening. The accuracy of this scale for identifying malnutrition is 98.7% [37]. The maximum total score was 14 points. A score of 12 or higher indicates a low risk of malnutrition; a score of 8–11 indicates a potential risk of malnutrition; and a score of 7 or below indicates a high risk of malnutrition.

#### Instrumental activities of daily living

We used the Instrumental Activities of Daily Living Scale (IADL) developed by Lawton and Brody (1969) to evaluate the participants’ activities of daily living [38]. This questionnaire covered going shopping, using transportation, preparing food, doing housekeeping and laundry, using the telephone, administering own medications, and managing own finances. The individual’s ability to perform each item was rated according to one of three levels: no ability (1 point), assistance required (2 points), or full independence (3 points). The total score ranged from 0 to 24 points.

#### Oral health-related quality of life (OHRQoL)

We used the Mandarin version of the short-form oral hygiene impact profile (OHIP-14T), translated and verified by Kuo (2011) [39], to determine the participants’ OHRQoL. According to previous studies, the internal consistency of the Mandarin version of OHIP-14T, as indicated by Cronbach’s alpha, is 0.862 for the seven dimensions and 0.882 for the 14 questions. The test–retest reliability intraclass correlation coefficient values are 0.86 for the seven dimensions and 0.835 for the 14 questions. The questionnaire consisted of 14 questions, each scoring a maximum of 4 points, so the total score ranged from 0 to 48 points.

### Data analysis

All data were analyzed using IBM SPSS statistical software (Additional file 1). For continuous variables we report means and standard deviations, and for categorical data we present numbers and percentages. We investigated the relationship between oral health literacy, OHRQoL, and gender, and therefore conducted subgroup analyses for each gender. OHRQoL was treated as a dependent variable, while oral health literacy was considered as an independent variable. The controlled variables (age, education level, monthly income, nutritional status, and instrumental activities of daily living) were included in the analyses. Statistical significance was considered at a two-sided *p* value of ≤ 0.05.

## Results

Of 216 surveys distributed, 14 surveys were deemed invalid (there were several unanswered questions, most of which were in the basic demographics section, followed by the oral health literacy section) and excluded. This left 202 questionnaires that were valid, yielding a 93.5% valid response rate.

Of the 202 participants, 114 (56.4%) were women and 88 (43.6%) were men, and the mean age was 74.72 years (SD = 8.17). For education level, 107 participants (53.0%) had elementary school education or below, and 95 (47.0%) had at least junior high education. Just under half (91, 45.0%) had no monthly income, leaving 111 (55.0%) participants who did have some monthly income (Table 1).

**Table 1:** Descriptive statistics of demographics, oral health literacy and OHRQoL (N = 202)

Variables	*n* (%)	Mean ± SD
Demographics		
Gender		
Female	114 (56.4)	
Male	88 (43.6)	
Age		74.72 ± 8.17
Educational level		
Elementary school	107 (53.0)	
Junior high school and above	95 (47.0)	
Individual monthly income		
No	91 (45.0)	
Yes	111 (55.0)	
Oral health literacy (MQHL-AQ)		9.39 ± 4.07
Reading comprehension (range 0–5)		2.10 ± 1.32
Numeracy (range 0–4)		2.12 ± 1.47
Listening skills (range 0–2)		1.12 ± 0.80
Decision-making (range 0–5)		3.11 ± 1.33
Number of insufficient literacy	92 (45.5)	
OHRQoL (OHIP-14 T)		6.96 ± 7.70
Functional limitation		0.48 ± 0.63
Physical pain		0.56 ± 0.69
Psychological discomfort		0.73 ± 0.79
Physical disability		0.50 ± 0.62
Psychological disability		0.46 ± 0.64
Social disability		0.33 ± 0.52
Handicap		0.44 ± 0.60

*SD* Standard Deviation, *MOHL-AQ* Mandarin version of the oral health literacy adult questionnaire, *OHRQoL* Oral health-related quality of life, *OHIP-14 T* Short-form Oral Hygiene Impact Profile Taiwanese version

### Oral health literacy

The participants’ mean oral health literacy score was 9.39 points (SD = 4.07) out of a maximum possible of 17 (Table 1). A total of 92 participants (45.5%) had a low level of oral health literacy, with a score of 9 or below. Forty-two (20.8%) had a medium level of oral health literacy (score of 10–11), while 68 (33.7%) had a sufficient level (score of 12–17; Table 2). Of all the questions in the oral health literacy questionnaire, Question 4 (*Continuing from the previous question, how many teeth of this kind are usually present at the age of six?*) had the lowest percentage of correct responses (8.9%), followed by Question 14 (28.2%) (*What do you think it means if the consent form says, “My dentist is exempt from responsibility for unintentional complications?”*).

**Table 2:** Factors associated with Oral Health-Related Quality of Life (OHR-QoL) (n = 114)

	Women (n = 114)				Men (n = 88)			
Factors	B	SE	Odds ratio	*p*-values	B	SE	Odds ratio	*p*-values
Oral health literacy	− 0.122	0.092	0.885	0.186	**0.593**	**0.215**	**1.809**	**0.006**
Age	− 0.058	0.038	0.944	0.126	− 0.034	0.056	0.967	0.542
Instrumental activities of daily living	− 0.072	0.049	0.930	0.139	− **0.286**	**0.096**	**0.751**	**0.003**
Nutrition	0.034	0.114	1.035	0.763	0.244	0.192	1.277	0.202
Education (ref: Elementary school)	0.824	0.754	2.281	0.274	− 0.646	0.877	0.524	0.461
Income (ref: no income)	− 1.125	0.737	0.325	0.127	0.019	0.987	1.019	0.985

Significant items in bold; Original OHRQoL score of 0 were considered to have no OHRQoL problems and coded as 1. Participants with an original score of ≥ 1 point were considered to have OHRQoL problems and were assigned a code of 0

We analyzed the oral health literacy data in four sections: reading comprehension (reading and knowledge skills); numeracy (reading, writing and calculation skills); listening skills (listening, reading, writing, calculation and communication skills); and appropriate decision-making (reading, comprehension, and decision-making skills). The score was highest for appropriate decision-making (mean = 3.11, SD = 1.33), followed by numeracy (mean = 2.12, SD = 1.32). It was lowest for listening skills (mean = 1.12, SD = 0.80) (Table 1).

### Oral health-related quality of life (OHRQoL)

The participants’ mean oral quality of life score was 6.96 points (SD = 7.70; Table 1). For the individual questions, the score was highest for the question about detecting tooth problems (mean = 0.76, SD = 0.86), followed by worries (mean = 0.69, SD = 0.83). The OHRQoL data were analyzed for each of the seven dimensions (functional limitation, physical pain, psychological discomfort, physical disability, psychological disability, social disability, and handicap). The psychological discomfort dimension had the highest score (mean = 0.73, SD = 0.79), followed by physical pain (mean = 0.56, SD = 0.69) (Table 1).

We conducted subgroup analyses according to gender, treating OHRQoL as a categorical variable. Those with an original OHRQoL score of 0 were considered to have no OHRQoL problems and coded as 1. Participants with an original score of ≥ 1 point were considered to have OHRQoL problems and were assigned a code of 0. We conducted logistic regression analyses to explore the relationship between the dependent variable (OHRQoL) and the independent variable (oral health literacy). The participants’ age, education level, average monthly income, nutritional status, and activities of daily living were controlled for in the statistical model. The relationship between OHRQoL and oral health literacy was significant for men (OR 1.809; *p* = 0.006, Table 2), but not for women (OR 0.885; *p* = 0.186).

## Discussion

Because this study focused only on community-dwelling older adults with a typical level of oral health literacy, we excluded participants with a high level of oral health literacy (12–17 points), based on the results of a previous study [40]. We recoded the OHRQoL score to aid assessment and interpretation. Based on the new codes, the higher the score, the better the OHRQoL.

We also converted the OHRQoL score into a categorical variable with only two categories: “0” (for all scores of ≥ 1) and “1” (for all scores of < 1). We found that when age, education level, average monthly income, nutritional status, and activities of daily living were controlled for, for a one-point increase in oral health literacy score in males, the OHRQoL increased by 0.593 points, indicating that the better male participants’ oral health literacy, the better the OHRQoL.

This relationship was not statistically significant for the female subgroup, however. Chi-squared test results revealed that education level and average monthly income differed according to gender. The fact that the relationship between oral health literacy and OHRQoL was only significant for males might therefore be attributed to gender differences in educational level and average monthly income. This might also highlight the inequities experience by women in terms of health education and awareness about good oral health practices.

Our results are in line with our original hypotheses. There was no significant relationship between women’s oral health literacy and their OHRQoL. However, good OHRQoL is an integral part of overall health, but it is affected by differences in oral health and the accessibility of medical services [41]. The US CDC (2021) recommended the following methods to maintain oral and dental health: brushing teeth with fluoride toothpaste, brushing teeth twice a day, flossing to remove plaque between teeth, visiting the dentist at least annually, not smoking, limiting intake of alcoholic beverages, keeping diabetes under control to avoid complications, and assisting elderly people who cannot function independently with their dental cleaning, using toothbrushes and dental floss [42].

Improving the oral health literacy of community-dwelling older adults can improve their OHRQoL. However, because the education level differed according to gender, we recommend that gender-specific oral health literacy education is offered through community health-education programs. Since the men’s education level was higher than the women’s, oral-health education for man could be provided using textbooks as teaching material. The women, however, may have more difficulty with reading, so we would recommend conducting their oral-health education using films in their native language to improve their oral health literacy and OHRQoL.

Several limitations to be acknowledged in this study. First, the use of convenience sample may limit the generalizability of the study result. Second, both oral health literacy and OHRQoL measures are self-reported, there may have potential social desirability bias exists. Therefore, the findings generated from this study should be interpreted with caution (Additional file 1).

## Conclusions

In this study. We found that the relationship between oral health literacy and OHRQoL was only significant for men. No significant relationship between women’s oral health literacy and their OHRQoL. However, good OHRQoL is an integral part of overall health, but it is influenced by differences in oral health and the accessibility of healthcare services. We recommend that future research be expanded to include every part of the country, to increase the sample size. In addition, objective assessment methods should be added, to improve the representativeness of the research results. This study can inform future research to design gender-specific health education programs in the future.

## Supplementary Information

**Additional file 1**. Dataset file.

## Data Availability

The datasets used and/or analysed during the current study are available from the corresponding author on reasonable request.

## Abbreviations

CDC: Centers for disease control and prevention
MNA-SF: Mini nutritional assessment-short form
IADL: Instrumental activities of daily living
OHRQoL: Oral health-related quality of life
OHL: Oral health literacy
OHL-AQ: Oral health literacy adult questionnaire
WHO: World Health Organization
