# Supplementary material for: Gender differences in the association between oral health literacy and oral health-related quality of life in older adults
Source: BMC Oral Health. 2022 May 25;22:205. doi: 10.1186/s12903-022-02237-8 (PMC9131657; doi:10.1186/s12903-022-02237-8)
Supplement: Supplementary file 1 — Additional file 1. Dataset file. [file 12903_2022_2237_MOESM1_ESM.pdf]

no	sex	birth	years	edu	New_edu	live	income	New_incon
1	1	10-Feb-52	69	2	2	3	1	1
2	0	10-Dec-37	83	1	1	3	1	1
3	1	16-Oct-55	65	4	4	3	1	1
4	1	2-Aug-52	68	4	4	2	2	2
5	1	10-May-49	71	3	3	3	2	2
6	0	23-Mar-53	67	3	3	3	1	1
7	0	1-Feb-51	70	2	2	3	1	1
8	0	20-Aug-41	79	4	4	3	2	2
9	0	15-Aug-47	73	4	4	3	2	2
10	1	19-Oct-51	69	6	6	2	3	3
11	0	30-May-47	73	2	2	3	2	2
12	0	30-Dec-47	73	5	5	3	2	2
13	1	16-Oct-55	65	3	3	3	2	2
14	1	1-Mar-48	73	2	2	3	1	1
15	0	29-Oct-52	68	5	5	2	1	1
16	0	26-Feb-52	69	2	2	3	1	1
17	0	1-Jun-39	81	2	2	3	1	1
18	0	19-Oct-51	69	5	5	3	1	1
19	1	26-Oct-38	82	2	2	3	1	1
20	0	6-Feb-56	65	4	4	2	4	3
21	1	12-Jul-47	73	3	3	3	2	2
22	1	16-Feb-54	67	5	5	3	3	3
23	1	19-Jan-51	70	4	4	3	4	3
24	0	14-Feb-56	65	4	4	3	3	3
25	0	23-Jul-37	83	2	2	3	1	1
26	0	20-Oct-47	73	2	2	3	1	1
27	1	16-Jul-24	96	4	4	3	1	1
28	1	13-Jun-51	69	5	5	2	3	3
29	0	9-Oct-49	71	6	6	2	3	3
30	1	2-Mar-52	69	5	5	1	2	2
31	0	26-Aug-47	73	4	4	3	2	2
32	0	31-Jan-51	70	6	6	2	4	3
33	0	29-Jan-53	68	5	5	3	3	3
34	1	4-Oct-51	69	4	4	3	3	3
35	0	1-Mar-42	79	1	1	3	2	2
36	1	24-Mar-55	65	4	4	2	3	3
37	0	28-Mar-54	66	4	4	3	2	2
38	1	28-May-48	72	4	4	3	2	2
39	0	10-Aug-53	67	2	2	3	2	2
40	0	5-Mar-54	66	6	6	3	3	3
41	0	1-Aug-52	68	3	3	3	1	1
42	1	14-Jun-51	69	4	4	3	2	2
43	0	1-Feb-52	69	4	4	3	3	3
44	0	22-May-55	65	5	5	2	1	1
45	1	17-Mar-54	66	6	6	3	3	3
46	0	8-May-46	74	2	2	1	2	2
47	0	12-Jan-42	79	6	6	2	4	3
48	0	13-Apr-43	77	3	3	2	1	1
49	1	11-Dec-55	65	3	3	3	2	2

50	0	20-Mar-55	65	5	5	3	3	3
51	0	15-Oct-47	73	5	5	2	3	3
52	0	1-Mar-40	81	2	2	3	3	3
53	1	12-Apr-53	67	5	5	3	3	3
54	1	28-Jun-48	72	5	5	3	3	3
55	0	21-Dec-48	72	5	5	3	3	3
56	0	2-Mar-52	69	2	2	3	1	1
57	1	4-May-54	66	6	6	2	2	2
58	1	23-Jul-37	83	4	4	2	3	3
59	0	2-Nov-39	81	2	2	2	1	1
60	0	29-Mar-52	68	4	4	4	3	3
61	1	20-Sep-54	66	4	4	3	3	3
62	1	17-Feb-51	70	3	3	3	3	3
63	0	2-Mar-53	68	2	2	3	3	3
64	0	7-Mar-55	65	4	4	2	1	1
65	1	29-Jan-40	81	5	5	2	4	3
66	0	27-Apr-41	79	4	4	2	3	3
67	0	20-Feb-39	82	3	3	2	2	2
68	0	20-May-48	72	6	6	2	3	3
69	1	11-Jul-48	72	5	5	1	3	3
70	1	1-Nov-54	66	5	5	2	3	3
71	1	26-Aug-46	74	6	6	3	3	3
72	0	6-Jan-29	92	1	1	3	2	2
73	0	24-Sep-47	73	3	3	3	3	3
74	1	23-Aug-52	68	5	5	2	2	2
75	1	24-Nov-47	73	5	5	2	1	1
76	1	18-Sep-51	69	3	3	3	3	3
77	0	8-Aug-28	92	1	1	3	1	1
78	0	18-Jan-56	65	6	6	3	4	3
79	0	21-Sep-54	66	6	6	3	3	3
80	0	7-Dec-50	70	7	6	3	4	3
81	0	24-May-44	76	5	5	3	2	2
82	1	7-Jul-37	83	6	6	3	1	1
83	1	31-Jul-40	80	5	5	3	1	1
84	0	17-Apr-35	85	2	2	3	1	1
85	1	19-Apr-49	71	6	6	3	3	3
86	0	28-Jul-51	69	3	3	3	1	1
87	1	15-Apr-46	74	2	2	3	3	3
88	0	25-Oct-48	72	3	3	3	3	3
89	0	30-Aug-52	68	4	4	1	3	3
90	1	27-Nov-45	75	7	6	2	3	3
91	0	17-Jan-56	65	6	6	1	3	3
92	0	20-Nov-45	75	2	2	3	4	3
93	0	2-Sep-35	85	5	5	3	3	3
94	1	2-Nov-36	84	6	6	3	3	3
95	1	9-Sep-50	70	6	6	2	4	3
96	0	26-Nov-51	69	4	4	3	3	3
97	1	12-Sep-41	79	3	3	2	3	3
98	1	1-Jul-31	89	6	6	2	2	2
99	1	10-Dec-48	72	6	6	3	4	3

100	0	6-Jan-39	82	2	2	3	2	2
101	1	3-Jan-50	71	2	2	2	3	3
102	0	31-Mar-52	68	2	2	2	1	1
103	1	1-Feb-41	80	2	2	2	2	2
104	0	21-May-55	65	6	6	3	3	3
105	1	27-Jul-54	66	4	4	3	3	3
106	1	14-Apr-36	84	2	2	1	1	1
107	0	20-Feb-45	76	2	2	2	2	2
108	0	30-May-54	66	5	5	5	3	3
109	0	3-Dec-51	69	4	4	3	4	3
110	0	10-Jul-50	70	5	5	2	4	3
111	1	27-Feb-48	73	5	5	2	2	2
112	1	8-Jul-38	82	2	2	3	1	1
113	1	4-Mar-55	65	5	5	3	3	3
114	1	12-Jul-42	78	3	3	2	2	2
115	1	27-May-41	79	6	6	2	4	3
116	0	19-Nov-46	74	2	2	2	2	2
117	1	8-Jan-51	70	3	3	3	3	3
118	0	19-Jun-55	65	4	4	3	1	1
119	0	20-Jun-48	72	6	6	2	2	2
120	0	27-May-51	69	5	5	3	3	3
121	0	24-Nov-46	74	6	6	3	3	3
122	1	19-Aug-44	76	5	5	3	1	1
123	0	29-Aug-37	83	6	6	3	1	1
124	0	10-Feb-44	77	2	2	3	1	1
125	1	15-Sep-50	70	4	4	1	3	3
126	1	26-Dec-50	70	5	5	2	2	2
127	1	19-Mar-54	66	6	6	3	3	3
128	0	30-Apr-47	73	3	3	3	1	1
129	1	26-Oct-48	72	1	1	2	1	1
130	1	3-Sep-54	66	2	2	2	2	2
131	0	21-Jun-43	77	2	2	2	1	1
132	0	22-Oct-51	69	3	3	2	1	1
133	0	1-Dec-23	97	1	1	3	1	1
134	0	9-Mar-32	88	1	1	5	1	1
135	0	12-Nov-49	71	1	1	3	1	1
136	0	16-Feb-42	79	2	2	2	1	1
137	0	1-Oct-49	71	2	2	2	1	1
138	1	12-Apr-44	76	6	6	2	1	1
139	1	24-Nov-44	76	2	2	3	2	2
140	1	4-Jul-43	77	2	2	2	2	2
141	1	31-Oct-54	66	6	6	1	3	3
142	0	5-Aug-46	74	1	1	2	1	1
143	0	15-Jun-27	93	2	2	3	1	1
144	0	3-Mar-23	97	1	1	3	1	1
145	0	20-Dec-12	108	1	1	3	2	2
146	0	16-Aug-24	96	4	4	3	1	1
147	0	20-May-52	68	1	1	3	1	1
148	1	10-Nov-34	86	2	2	2	1	1
149	1	8-Feb-27	94	3	3	2	1	1

150	0	12-Jan-41	80	2	2	2	1	1
151	0	17-Jun-37	83	2	2	2	1	1
152	0	4-Oct-36	84	2	2	3	1	1
153	1	15-Jun-31	89	2	2	2	1	1
154	0	11-Jan-45	76	1	1	3	1	1
155	0	21-Aug-27	93	4	4	3	1	1
156	1	2-Jun-53	67	3	3	2	2	2
157	0	28-Jun-50	70	2	2	3	1	1
158	0	22-Feb-49	72	3	3	2	1	1
159	1	30-Jan-41	80	1	1	2	1	1
160	0	4-Sep-55	65	4	4	2	2	2
161	1	8-Oct-43	77	2	2	2	2	2
162	1	2-Dec-50	70	2	2	3	1	1
163	0	16-Jun-44	76	5	5	3	2	2
164	0	2-Oct-52	68	2	2	2	3	3
165	0	10-Jul-49	71	4	4	2	2	2
166	1	27-Aug-46	74	3	3	2	1	1
167	0	15-May-49	71	2	2	2	1	1
168	1	7-May-51	69	2	2	1	2	2
169	0	9-Feb-41	80	2	2	3	1	1
170	0	25-Sep-50	70	2	2	2	1	1
171	1	9-Nov-42	78	2	2	2	1	1
172	1	10-Oct-42	78	2	2	2	1	1
173	0	9-Oct-52	68	2	2	2	2	2
174	1	27-Aug-46	74	3	3	2	1	1
175	1	13-Apr-39	81	2	2	3	1	1
176	0	15-May-49	71	2	2	2	1	1
177	0	16-Dec-41	79	2	2	2	1	1
178	0	25-Oct-49	71	2	2	3	1	1
179	0	20-Mar-49	71	2	2	3	1	1
180	0	7-Apr-40	80	1	1	3	1	1
181	1	1-Sep-47	73	3	3	2	1	1
182	0	1-Mar-36	85	2	2	3	1	1
183	0	16-Oct-45	75	3	3	2	1	1
184	1	12-Feb-26	95	6	6	2	1	1
185	1	10-Jan-46	75	6	6	2	1	1
186	0	20-May-52	68	1	1	3	1	1
187	1	2-Dec-37	83	3	3	2	1	1
188	1	1-Mar-34	87	5	5	2	1	1
189	0	5-Dec-52	68	3	3	2	2	2
190	1	2-Apr-49	71	4	4	2	1	1
191	0	6-Feb-36	85	1	1	2	1	1
192	1	1-Apr-32	88	4	4	2	1	1
193	1	3-Jun-47	73	2	2	2	1	1
194	0	2-Feb-42	79	2	2	2	1	1
195	0	20-Sep-29	91	1	1	3	1	1
196	0	2-Aug-41	79	1	1	3	1	1
197	0	20-Feb-24	97	1	1	3	1	1
198	0	28-Jan-39	82	1	1	3	1	1
199	1	23-Jan-55	66	2	2	2	2	2

200	1	7-Nov-47	73	6	6	2	1	1
201	1	5-Mar-49	72	4	4	2	2	2
202	1	27-Jul-38	82	6	6	3	2	2

work	history_1	history_2	history_3	history_4	history_5	history_6	history_7	BH
1	1	0	0	0	0	0	0	162.0
1	1	1	0	0	0	0	0	148.0
1	0	0	0	0	0	0	0	156.0
1	1	0	0	0	0	0	0	162.0
1	1	0	0	0	0	0	0	170.0
1	0	0	0	0	0	0	0	158.0
1	0	1	0	0	0	0	0	152.0
2	0	0	0	1	0	0	0	157.0
1	1	1	0	0	0	0	0	153.0
2	0	0	0	1	0	0	0	169.0
1	1	0	0	0	0	0	0	154.0
1	0	0	0	0	1	0	0	150.0
1	1	0	0	0	0	0	0	165.0
1	1	0	0	0	0	0	0	163.0
1	0	0	0	0	0	0	0	152.0
1	1	0	0	0	0	0	0	157.0
1	0	0	1	0	0	0	0	153.0
1	0	0	0	0	0	1	0	155.0
1	1	0	0	0	1	0	0	163.0
2	0	0	0	0	0	0	0	170.0
1	1	0	0	0	0	0	0	168.0
2	0	1	0	0	0	0	痛風	170.0
1	0	0	0	0	0	0	0	160.0
1	0	0	0	0	0	0	0	145.0
1	0	0	0	0	0	0	心臟病	145.0
1	1	0	0	1	0	1	0	150.0
1	1	0	0	0	0	0	帕金森氏[illegible]	160.0
1	0	0	0	1	0	0	0	170.0
1	1	0	0	0	0	0	0	155.0
1	0	0	0	0	0	0	0	170.0
1	0	0	0	0	0	0	0	160.0
1	0	0	0	1	0	0	0	155.0
1	0	0	0	0	0	0	0	150.0
1	0	0	0	0	0	0	0	178.0
1	1	0	0	0	0	0	0	147.0
1	1	1	0	0	0	0	0	170.0
2	0	0	0	0	0	0	0	158.0
1	0	0	0	0	0	0	0	158.0
1	1	1	0	1	0	0	0	154.0
1	0	0	0	0	0	0	0	158.0
2	0	0	0	0	0	0	0	150.0
1	1	0	0	0	0	0	0	150.0
2	1	0	0	0	0	0	膝蓋骨刺	160.0
1	0	0	0	0	0	0	0	156.0
1	0	0	0	0	0	0	0	180.0
1	0	0	0	0	0	0	0	140.0
1	1	0	0	1	0	0	0	175.0
1	0	0	0	0	0	1	0	160.0
1	0	0	0	0	0	1	0	163.0

1	1	0	0	0	0	0	0	155.0
1	0	0	0	0	0	0	0	156.0
1	0	0	0	1	0	0	0	156.0
1	1	0	0	0	0	0	0	167.0
1	1	0	0	0	0	0	0	163.0
1	0	0	0	0	0	0	高膽固醇	152.0
1	0	0	0	0	0	0	0	150.0
1	1	0	0	0	0	0	0	166.0
1	1	0	0	0	0	0	0	154.0
1	1	0	0	0	0	0	0	156.0
1	1	0	0	0	0	0	0	158.0
2	0	1	0	0	0	0	0	158.0
1	0	0	0	1	0	0	0	170.0
1	1	0	0	0	0	0	0	163.0
1	1	0	0	0	0	0	0	156.0
1	1	1	1	1	0	0	0	182.0
1	0	1	0	0	0	0	0	161.0
1	0	0	0	1	0	0	乳癌	148.5
1	0	0	0	0	0	0	乳癌	157.0
1	1	1	0	0	0	0	0	178.0
1	0	0	0	0	0	0	0	164.0
1	1	0	0	0	0	0	0	163.0
1	1	0	0	0	0	0	0	157.0
1	1	0	0	0	0	0	心臟病	156.0
1	1	0	0	0	0	0	0	178.0
1	0	1	0	1	0	0	0	169.0
1	1	0	0	0	0	1	0	166.0
1	1	1	0	0	0	0	0	160.0
1	0	0	0	0	0	0	淋巴血管	154.0
1	0	0	0	1	0	0	地中海型	155.5
1	0	0	0	0	0	0	0	162.0
1	0	0	0	0	0	1	0	155.0
1	0	0	0	1	0	0	0	168.0
1	1	0	0	1	0	0	0	164.0
1	1	1	0	1	0	0	0	157.0
1	0	0	0	0	0	0	0	165.0
1	0	0	0	1	0	0	胃痛	152.0
1	0	0	0	0	0	0	0	162.0
1	1	0	0	0	0	0	0	155.0
2	1	1	0	0	0	0	0	154.0
1	1	0	0	0	0	1	0	158.0
1	0	0	0	0	0	0	0	160.0
1	1	0	0	0	0	0	0	158.0
1	1	0	0	1	0	0	0	156.0
1	0	0	0	0	0	0	0	158.0
1	1	0	0	0	0	0	0	167.0
1	0	0	0	1	0	0	0	160.0
1	1	0	0	1	0	0	肝硬化	168.0
1	0	0	0	0	0	0	帕金森氏	165.0
1	0	0	0	1	0	0	0	168.0

1	1	0	0	1	1	0	0	145.0
2	0	0	0	0	0	1	0	162.0
1	0	0	0	0	0	1	0	161.0
1	0	0	0	0	0	0	0	170.0
2	0	0	0	0	0	0	0	156.0
1	0	0	0	0	0	0	0	178.0
1	1	0	0	1	0	0	0	170.0
1	1	0	0	0	0	1	皮膚	145.0
1	0	1	0	0	0	0	0	154.0
1	0	0	0	0	0	0	膽固醇稍高	154.0
1	0	1	0	1	0	0	0	165.0
1	0	0	0	1	0	0	0	168.0
1	1	0	0	1	0	0	0	171.0
1	0	0	0	0	0	0	0	168.0
1	0	0	0	1	0	0	0	170.0
1	1	0	0	0	0	0	0	158.0
1	0	0	0	1	0	1	0	160.0
1	1	0	0	0	0	0	0	171.0
1	0	0	0	0	0	1	0	157.0
1	0	0	0	1	0	1	0	150.0
1	0	0	0	0	0	1	0	166.0
2	0	0	0	0	0	0	0	150.0
1	1	0	0	0	0	0	0	169.0
1	0	0	0	0	0	1	0	158.0
1	0	1	0	0	0	0	0	152.0
1	0	0	0	0	0	1	0	172.0
1	1	0	0	1	0	0	0	173.0
2	1	0	0	0	0	1	0	165.0
1	0	0	0	1	0	0	0	153.0
1	0	0	0	0	0	0	癌症	161.0
1	0	0	0	0	0	1	帕金森氏病	170.0
1	0	0	0	0	0	1	0	160.0
1	1	0	0	0	0	0	0	163.0
1	0	0	0	0	0	0	失智症	145.0
1	1	1	0	0	0	0	0	141.0
1	0	1	0	0	0	0	0	148.0
1	1	0	0	0	0	1	0	152.0
1	0	0	0	0	1	0	0	154.0
1	0	1	0	0	1	0	癌症	170.0
1	0	0	0	1	0	1	0	167.0
1	1	0	0	0	0	0	心臟病	170.0
1	0	0	1	0	0	0	痛風、肝病	167.0
1	0	1	0	1	0	0	0	150.0
1	1	0	0	0	0	0	心臟病	156.0
1	1	0	0	1	0	1	失智症	150.0
2	0	0	0	0	0	1	0	148.0
1	0	0	0	1	0	0	0	148.0
1	1	1	0	0	0	0	0	155.0
1	0	1	0	0	0	0	失智症	160.0
1	1	0	0	0	0	0	0	168.0

1	0	0	0	1	1	0	甲狀腺腫	158.0
1	1	0	0	0	0	0	0	148.0
1	1	1	0	1	0	1	0	153.0
1	1	0	0	0	0	0	0	167.0
1	0	1	0	0	1	0	0	155.0
1	1	0	0	0	0	0	0	143.0
1	1	1	0	0	0	0	癌症	162.0
1	1	1	0	0	0	0	0	148.0
1	1	0	0	1	1	0	0	148.0
1	0	0	0	0	0	1	帕金森氏[illegible]	158.0
1	0	0	0	0	0	0	心臟病	159.0
1	1	1	0	0	0	0	0	155.0
1	0	1	0	1	0	0	0	152.0
1	0	0	0	1	0	1	0	143.0
2	1	1	0	0	0	0	0	152.0
1	0	0	0	0	0	0	心臟病	151.0
1	1	0	0	0	0	0	0	170.0
1	0	1	0	0	0	0	0	160.0
1	1	1	0	0	0	0	0	168.0
1	0	0	0	0	0	0	癲癇	150.0
1	0	1	0	0	1	0	0	153.0
1	1	1	0	0	0	0	0	170.0
1	0	1	0	0	0	0	0	160.0
1	0	0	0	0	1	0	0	150.0
1	1	0	0	0	0	0	0	170.0
1	0	0	0	0	0	0	白血病	156.0
1	0	1	0	0	0	0	0	160.0
1	1	0	0	0	0	1	0	156.0
1	1	0	0	1	0	0	0	154.0
1	1	1	0	0	0	0	0	146.0
1	0	0	0	0	0	1	0	150.0
1	0	0	0	0	0	0	癌症	170.0
1	0	1	0	0	0	0	失智症	160.0
1	0	0	0	0	0	1	帕金森氏[illegible]	158.0
1	0	0	0	1	0	0	0	162.0
1	1	0	0	0	0	0	心臟病	160.0
1	1	1	0	0	0	0	0	155.0
1	1	1	0	0	0	0	高膽固醇[illegible]	174.0
1	1	0	0	1	1	1	心臟病	167.0
1	1	1	0	0	1	0	0	158.0
1	0	1	0	0	0	0	0	170.0
1	1	0	0	0	1	0	0	150.0
1	1	0	0	0	1	0	0	160.0
1	1	0	0	0	1	0	痛風	164.0
1	1	1	0	0	1	0	0	155.0
1	0	0	0	0	0	1	癌症	151.0
1	0	0	0	0	0	1	0	145.0
1	1	0	0	0	0	0	心臟病	150.0
1	1	0	0	0	0	0	高血脂	156.0
1	1	1	0	0	0	0	痛風	172.0

1	0	0	0	0	0	0	0	191.0
1	1	1	0	0	0	0	0	168.0
1	1	1	0	0	0	0	癌症	172.0

BW	BMI	smoking	betelnut	tool_1	tool_2	tool_3	tool_4	tool_5
65.0	24.77	1	2	1	1	0	1	0
48.0	21.91	1	1	1	1	0	1	0
69.0	28.35	1	1	1	1	0	0	0
48.0	18.29	1	1	1	1	0	0	0
72.0	24.91	1	1	1	0	0	1	0
60.0	24.03	1	1	1	0	0	1	0
56.0	24.24	1	1	1	1	0	0	0
59.0	23.94	1	1	1	0	0	1	0
50.0	21.36	1	1	1	1	0	1	0
58.0	20.31	1	1	1	1	1	0	0
50.0	21.08	1	1	1	0	0	0	0
50.0	22.22	1	1	1	0	0	0	0
57.0	20.94	1	1	1	1	0	0	0
60.0	22.58	1	1	1	0	0	1	0
53.0	22.94	1	1	1	1	0	1	0
58.0	23.53	1	1	1	1	0	1	0
65.0	27.77	1	1	1	1	0	0	0
46.0	19.15	1	1	1	1	0	0	0
49.0	18.44	1	1	1	1	0	0	0
60.0	20.76	1	1	1	0	0	1	0
68.0	24.09	1	1	1	0	0	1	0
86.0	29.76	2	2	0	0	0	0	0
55.0	21.48	1	1	1	0	0	1	0
58.0	27.59	1	1	1	0	0	1	0
54.0	25.68	1	1	1	0	0	0	0
40.0	17.78	1	1	1	0	0	0	0
42.0	16.41	1	1	1	0	0	0	0
68.0	23.53	1	1	1	0	1	1	0
59.0	24.56	1	1	1	0	1	0	0
80.0	27.68	1	1	1	1	0	0	0
60.0	23.44	1	1	1	1	1	1	0
55.0	22.89	1	1	1	0	0	1	0
50.0	22.22	1	1	1	0	1	0	0
62.0	19.57	1	1	1	1	0	1	0
52.0	24.06	1	1	1	0	0	0	0
86.0	29.76	1	1	1	0	0	0	0
54.0	21.63	1	1	1	0	1	1	0
55.0	22.03	2	1	1	0	0	0	0
57.0	24.03	1	1	1	1	0	0	0
64.0	25.64	1	1	1	0	0	0	0
60.0	26.67	1	1	1	0	0	0	0
47.0	20.89	1	1	1	0	0	1	0
68.0	26.56	1	1	1	0	0	1	0
56.0	23.01	1	1	1	1	0	1	0
77.0	23.77	2	1	1	0	0	0	0
51.0	26.02	1	1	1	0	0	0	0
74.0	24.16	1	1	1	0	1	0	0
58.0	22.66	1	1	0	0	1	0	0
50.0	18.82	1	1	1	0	0	0	0

54.0	22.48	1	1	1	0	1	0	0
56.0	23.01	1	1	1	0	0	0	0
57.0	23.42	1	1	1	0	0	0	0
71.0	25.46	1	1	1	1	0	0	0
60.0	22.58	1	1	1	0	1	0	0
54.0	23.37	1	1	1	1	1	0	0
65.0	28.89	1	1	1	0	0	0	0
75.0	27.22	1	1	1	0	1	0	0
68.0	28.67	1	1	1	0	0	0	0
64.0	26.30	1	1	1	0	0	0	0
62.0	24.84	1	1	1	0	0	1	0
52.0	20.83	1	1	1	0	0	0	0
72.0	24.91	1	1	1	0	0	1	0
58.5	22.02	1	1	1	0	0	0	0
58.0	23.83	1	1	1	0	0	0	0
80.0	24.15	1	1	1	0	1	0	0
55.0	21.22	1	1	1	0	1	0	0
48.0	21.77	1	1	1	0	1	0	0
64.0	25.96	1	1	1	1	1	0	0
78.0	24.62	1	1	1	0	0	0	0
68.0	25.28	1	1	1	0	0	1	0
85.0	31.99	1	1	1	0	0	1	0
60.0	24.34	1	1	1	0	0	0	0
63.0	25.89	1	1	1	1	1	0	0
65.0	20.52	1	1	1	0	0	0	0
65.0	22.76	1	1	1	0	0	1	0
78.0	28.31	1	1	1	0	0	0	0
65.0	25.39	1	1	1	0	0	0	0
61.0	25.72	1	1	1	1	1	1	0
49.0	20.26	1	1	1	1	1	0	0
60.0	22.86	1	1	1	1	0	0	0
50.0	20.81	1	1	1	1	0	0	0
60.0	21.26	1	1	1	1	0	0	0
65.0	24.17	1	1	1	0	1	1	0
53.0	21.50	1	1	1	0	0	0	0
65.0	23.88	1	1	1	0	1	0	0
56.0	24.24	1	1	1	0	0	1	0
55.0	20.96	1	1	1	0	0	0	0
60.0	24.97	1	1	1	1	0	0	0
49.0	20.66	1	1	1	1	0	0	0
57.0	22.83	1	1	1	1	0	0	0
51.0	19.92	1	1	1	1	0	0	0
62.0	24.84	1	1	1	0	0	1	0
63.0	25.89	1	1	0	1	0	1	0
60.0	24.03	1	1	1	0	0	0	0
78.0	27.97	2	1	1	0	0	0	0
51.0	19.92	1	1	1	0	0	0	0
69.0	24.45	1	1	1	0	0	0	0
45.0	16.53	1	1	1	0	0	0	0
58.0	20.55	2	1	1	0	0	0	0

53.0	25.21	1	1	1	0	0	0	0
72.0	27.43	1	1	1	0	0	0	0
64.0	24.69	1	1	1	1	0	0	0
65.0	22.49	1	1	1	0	0	0	0
60.0	24.65	1	1	1	0	1	0	0
95.0	29.98	1	1	1	0	1	0	0
65.0	22.49	1	1	1	0	0	0	0
55.0	26.16	1	1	1	1	0	0	0
59.0	24.88	1	1	1	0	0	0	0
55.0	23.19	1	1	1	0	0	1	0
58.0	21.30	1	1	1	1	1	0	0
68.0	24.09	1	1	1	0	0	0	0
75.0	25.65	1	1	1	0	0	0	0
77.0	27.28	1	1	0	0	0	0	0
80.0	27.68	1	1	1	0	0	0	0
59.0	23.63	1	1	1	0	0	0	0
53.0	20.70	1	1	1	0	1	0	0
63.0	21.55	1	1	1	0	0	0	0
51.0	20.69	1	1	1	0	0	0	0
48.0	21.33	1	1	1	1	0	0	0
62.0	22.50	1	1	1	0	0	0	0
56.0	24.89	1	1	1	0	0	1	0
64.0	22.41	1	1	1	0	1	0	0
58.0	23.23	1	1	1	0	0	1	0
58.0	25.10	1	1	1	0	0	0	0
64.0	21.63	1	1	1	0	0	1	0
70.0	23.39	1	1	1	0	0	1	0
57.0	20.94	1	1	1	0	1	0	0
53.0	22.64	1	1	1	0	0	0	0
50.0	19.29	1	1	1	0	0	0	0
48.0	16.61	1	1	1	0	0	0	0
45.0	17.58	1	1	1	0	0	0	0
60.0	22.58	1	1	1	0	0	0	0
33.0	15.70	1	1	1	0	0	0	0
64.0	32.19	1	1	1	0	0	0	0
65.0	29.67	1	1	1	0	0	0	0
52.0	22.51	1	1	1	0	0	0	0
58.0	24.46	1	1	1	0	0	0	0
57.0	19.72	1	1	1	0	0	0	0
70.0	25.10	1	1	1	0	0	0	0
70.0	24.22	1	1	1	0	0	0	0
65.0	23.31	1	1	1	0	0	0	0
68.0	30.22	1	1	1	0	0	0	0
54.0	22.19	1	1	1	0	0	0	0
52.0	23.11	1	1	1	0	0	0	0
38.0	17.35	1	1	1	0	0	0	0
55.0	25.11	1	1	1	0	0	0	0
58.0	24.14	1	1	1	0	0	0	0
49.0	19.14	1	1	1	0	0	0	0
49.0	17.36	1	1	1	0	0	0	0

38.0	15.22	1	1	1	0	0	0	0
37.0	16.89	1	1	1	0	0	0	0
60.0	25.63	1	1	1	0	0	0	0
59.0	21.16	1	1	1	0	0	0	0
70.0	29.14	1	1	1	0	0	0	0
58.0	28.36	1	1	1	0	0	0	0
46.7	17.79	2	2	1	0	0	0	0
40.0	18.26	1	1	1	0	0	0	0
60.0	27.39	1	1	1	0	0	0	0
69.0	27.64	1	1	1	0	0	0	0
40.0	15.82	1	1	1	0	0	0	0
68.0	28.30	1	1	1	0	0	0	0
46.0	19.91	1	1	1	0	0	0	0
43.0	21.03	1	1	1	0	0	0	0
49.0	21.21	1	1	1	0	0	1	0
41.0	17.98	1	1	1	0	0	0	0
63.0	21.80	1	1	1	0	0	0	0
48.0	18.75	1	1	1	0	0	0	0
60.0	21.26	2	1	1	0	0	0	0
44.0	19.56	1	1	1	0	0	0	0
66.0	28.19	1	1	1	0	0	0	0
78.0	26.99	1	1	1	0	0	0	0
40.0	15.63	1	1	1	0	0	0	0
58.0	25.78	1	1	1	0	0	0	0
63.0	21.80	1	1	1	0	0	0	0
52.0	21.37	1	1	1	0	0	0	0
48.0	18.75	1	1	1	0	0	0	0
47.0	19.31	1	1	1	0	0	0	0
75.0	31.62	1	1	1	0	0	0	0
48.0	22.52	1	1	1	0	0	0	0
45.0	20.00	1	1	1	0	0	0	0
57.0	19.72	1	1	1	0	0	0	0
50.0	19.53	1	1	1	0	0	0	0
49.0	19.63	1	1	1	0	0	0	0
52.0	19.81	1	1	1	0	0	0	0
70.0	27.34	1	1	1	0	0	0	0
58.0	24.14	1	1	1	0	0	0	0
82.0	27.08	1	1	1	0	0	0	0
71.0	25.46	1	1	1	0	0	0	0
60.0	24.03	1	1	1	0	0	0	0
50.0	17.30	1	1	1	0	0	0	0
61.0	27.11	1	1	1	0	0	0	0
60.0	23.44	1	1	1	0	0	0	0
64.0	23.80	1	1	1	0	0	0	0
68.0	28.30	1	1	1	0	0	0	0
40.0	17.54	1	1	1	0	0	0	0
48.0	22.83	1	1	1	0	0	0	0
50.0	22.22	1	1	1	0	0	0	0
57.0	23.42	1	1	1	0	0	0	0
60.0	20.28	1	1	1	0	0	0	0

90.0	24.67	1	1	1	0	0	0	0
69.0	24.45	1	1	1	0	0	0	0
75.0	25.35	1	1	1	0	0	0	0

tool_6	tool_7	tool_8	tool_9	tool_10	tool_11	toolchoose	missingtee	denture
0	1	0	0	1	0	1	2	2
0	1	0	0	0	0	1	2	2
0	1	0	0	0	0	1	1	2
0	0	1	0	0	0	1	2	2
0	1	0	0	0	0	1	2	2
0	1	0	0	1	0	1	2	3
0	1	0	0	0	0	1	2	4
0	1	0	0	1	0	2	2	3
0	0	0	0	1	0	2	2	4
1	0	0	0	0	0	2	2	3
1	0	0	0	1	0	1	2	2
1	0	0	0	0	0	1	1	3
0	1	0	0	1	0	1	1	1
0	1	0	0	0	0	2	2	2
0	0	0	0	0	0	1	1	1
0	1	0	0	0	0	1	2	2
0	0	0	0	1	0	1	2	3
0	1	0	0	0	0	2	2	4
0	0	0	0	0	0	1	2	4
0	0	0	0	1	0	1	2	2
0	0	0	0	0	0	1	2	2
0	0	0	0	1	0	1	2	1
0	0	0	0	0	0	2	1	4
0	1	0	0	0	0	2	1	2
0	0	0	0	0	0	1	2	1
0	0	0	0	0	0	1	2	1
1	0	0	0	0	0	1	2	1
0	0	0	1	1	0	1	2	4
0	0	0	0	0	0	1	1	2
0	0	0	0	1	0	1	1	2
0	1	1	1	1	0	1	2	2
0	1	0	0	1	0	2	2	2
1	0	0	0	0	鹽水	1	1	2
0	1	0	0	0	開水	1	2	1
0	1	0	0	0	0	2	1	1
0	0	0	0	0	0	1	2	3
0	0	0	0	0	0	2	2	2
0	0	0	0	0	0	1	2	3
0	0	0	0	0	0	1	2	3
0	1	0	0	0	0	1	1	1
0	0	0	0	0	0	2	1	1
0	0	0	0	1	牙膏	2	1	2
1	1	0	0	0	開水	1	2	2
0	0	0	0	0	0	2	2	4
0	1	0	0	0	0	2	2	2
1	0	0	0	0	0	1	2	3
0	0	0	0	0	0	1	2	2
1	0	0	1	1	0	1	1	2
0	1	0	0	1	0	1	2	1

0	1	0	0	0	0	1	2	2
0	0	0	1	1	0	1	2	2
0	0	0	0	1	0	2	2	3
0	1	0	0	1	0	1	2	2
0	0	1	0	0	0	2	2	2
0	0	1	0	0	0	1	1	2
0	0	1	0	1	0	2	1	4
1	0	0	0	0	0	2	1	1
0	1	0	0	0	0	2	1	3
0	0	0	0	0	0	2	1	3
0	0	0	0	0	鹽水	2	2	2
0	0	0	0	0	0	1	2	3
0	0	0	0	0	0	2	2	1
0	1	0	1	0	0	2	1	2
0	0	0	0	1	0	1	1	2
0	1	0	0	0	0	1	2	4
0	0	0	0	0	0	2	2	2
0	0	0	0	0	0	1	1	2
0	0	0	0	1	0	1	1	4
0	0	0	1	1	0	2	2	2
0	0	0	0	0	0	1	1	1
0	0	0	0	0	0	2	2	1
0	1	0	0	0	0	1	1	4
1	0	0	1	1	0	2	2	2
0	1	0	0	0	0	1	2	3
0	0	0	0	0	0	1	2	2
0	0	0	0	0	0	1	2	1
0	0	0	0	0	0	1	2	1
1	1	1	1	1	0	1	1	2
1	0	0	0	0	0	2	1	2
0	0	0	0	0	0	2	2	1
0	0	0	0	0	0	2	2	3
0	0	0	0	0	0	2	2	1
0	0	0	0	0	0	1	2	2
0	0	0	0	1	0	1	1	3
0	0	0	0	0	0	2	1	1
0	0	0	0	0	0	1	1	2
0	0	0	0	0	0	1	2	2
0	1	0	0	0	0	1	2	2
0	0	0	0	0	0	2	2	2
0	0	0	0	0	0	1	2	4
0	0	0	0	0	0	2	1	1
1	0	0	0	0	0	1	2	4
0	0	0	0	1	0	1	2	2
0	0	1	0	1	0	1	1	4
0	0	0	0	0	0	1	2	3
0	1	0	0	0	0	1	1	1
0	0	0	0	0	自來水	1	2	4
0	0	0	0	0	0	1	2	1
0	0	0	0	0	溫鹽水	1	2	4

0	1	0	0	0	0	1	2	3
0	1	0	0	1	0	1	2	4
0	1	0	1	1	0	2	1	4
0	0	0	1	1	0	1	2	3
0	0	0	0	0	0	1	2	2
0	1	0	0	1	0	1	2	2
0	1	0	0	0	0	1	2	3
0	0	0	0	0	0	2	1	2
0	0	0	0	1	0	1	2	2
0	1	0	0	0	0	2	1	2
0	0	0	0	0	0	1	2	2
1	0	0	0	0	0	1	2	3
0	0	0	0	0	0	1	2	3
0	1	0	0	1	沖牙機	2	1	2
0	0	0	0	0	0	1	2	3
0	0	0	0	0	0	1	1	1
0	0	1	0	0	0	1	1	2
0	0	0	0	1	0	2	2	2
0	1	0	0	0	0	1	1	1
0	0	0	0	0	0	1	2	3
0	0	0	0	0	0	1	2	1
0	0	0	0	0	0	1	2	2
0	0	0	0	0	0	1	1	2
0	0	0	0	1	0	2	2	2
0	1	0	0	0	0	1	2	2
0	1	0	0	0	0	1	2	3
0	0	0	0	1	0	2	2	2
0	0	0	0	0	0	1	1	1
0	1	0	0	0	0	1	2	2
0	0	0	0	0	0	1	2	1
0	0	0	0	0	0	1	1	1
0	0	0	0	1	0	1	2	2
0	0	0	0	0	0	1	1	1
0	0	0	0	0	0	1	2	4
0	0	0	0	0	0	1	2	4
0	0	0	0	0	0	1	2	3
0	0	0	0	0	0	1	2	2
0	0	0	0	0	0	1	2	2
0	0	0	0	0	0	1	2	1
0	0	0	0	0	0	1	2	2
0	0	0	0	0	0	1	2	2
0	0	0	0	0	0	1	1	1
0	0	0	0	0	0	2	2	2
0	0	0	0	0	0	1	2	1
0	0	0	0	0	0	1	2	3
0	0	0	0	0	0	1	2	1
0	0	0	0	0	0	1	2	2
0	0	0	0	0	0	1	2	1
0	0	0	0	0	0	1	2	1
0	0	0	0	0	0	1	2	2

1	0	0	0	1	0	1	2	1
0	0	0	0	0	0	1	2	1
0	0	0	0	0	0	1	2	1
1	0	0	0	0	0	1	2	3
0	0	0	0	0	0	1	2	1
1	0	0	0	1	0	2	2	4
0	0	0	0	0	0	1	2	1
0	0	0	0	0	0	1	2	1
0	0	0	0	0	0	1	2	1
0	0	0	0	0	0	1	2	1
0	0	0	0	0	0	1	1	1
0	0	0	0	0	0	1	2	1
0	0	0	0	0	0	1	2	1
0	0	0	0	0	0	1	2	1
0	0	0	0	0	0	1	1	1
0	0	0	0	0	0	1	2	1
0	1	0	0	0	0	1	2	4
0	0	0	0	0	0	1	1	1
0	1	0	0	0	0	1	1	1
0	0	0	0	0	0	1	2	4
0	0	0	0	0	0	1	2	1
0	0	0	0	0	0	1	2	1
0	0	0	0	0	0	1	2	2
0	0	0	0	0	0	1	2	1
0	0	0	0	0	0	1	2	1
1	0	0	0	1	0	1	2	4
0	0	0	0	0	0	1	2	1
0	0	0	0	0	0	1	2	1
0	0	0	0	0	0	1	2	1
0	0	0	0	0	0	1	2	1
0	0	0	0	0	0	1	2	3
0	0	0	0	0	0	1	2	1
0	0	0	0	0	0	1	2	3
0	0	0	0	1	0	1	2	4
0	0	0	0	0	0	1	2	1
0	0	0	0	0	0	1	2	1
0	0	0	0	0	0	1	1	1
0	0	0	0	0	0	1	2	1
0	0	0	0	0	0	1	2	1
0	0	0	0	0	0	1	2	1
0	0	0	0	0	0	1	2	3
0	0	0	0	0	0	1	2	1
0	0	0	0	0	0	1	2	1
0	0	0	0	0	0	1	2	1
0	0	0	0	0	0	1	2	1
0	0	0	0	0	0	1	2	1
0	0	0	0	0	0	1	2	3
0	0	0	0	0	0	1	2	1
0	0	0	0	0	0	1	2	1
0	0	0	0	0	0	1	1	1

0	0	0	0	0	0	1	2	1
0	0	0	0	0	0	1	2	1
0	0	0	0	0	0	1	2	1

behavior_1	behavior_2	behavior_3	behavior_4	behavior_5	behavior_6	behavior_7	behavior_8	behavior_9
1	1	1	0	1	1	1	1	1
1	1	1	1	1	1	0	0	1
1	1	1	1	1	1	1	1	1
1	1	0	0	0	1	1	1	1
1	1	1	1	1	1	1	1	1
1	1	1	1	1	1	1	1	1
0	0	0	0	1	0	0	0	1
1	1	0	0	1	1	1	1	1
1	1	1	1	1	1	1	1	1
1	1	1	1	1	1	1	1	1
0	1	0	1	1	0	0	1	1
1	1	1	1	0	1	1	0	1
0	1	0	0	1	1	0	1	0
0	0	1	0	1	1	1	1	0
1	1	1	0	0	1	0	1	1
0	0	1	0	1	1	0	1	1
0	1	1	0	0	1	1	0	0
1	1	1	1	1	1	1	1	1
1	1	0	0	0	0	1	1	1
1	1	1	1	1	1	1	1	0
1	1	0	1	0	1	1	0	0
0	0	0	0	0	0	0	0	0
1	1	1	1	1	1	1	1	1
1	1	1	1	1	1	1	1	1
1	1	0	1	1	0	1	0	1
1	1	0	1	1	0	1	0	1
1	1	0	0	1	0	1	0	1
1	1	1	1	1	1	0	1	1
0	1	1	1	0	1	1	0	0
1	1	1	1	1	1	1	0	0
1	1	1	1	1	1	1	1	1
1	1	1	0	1	1	1	1	0
1	1	1	0	1	1	1	0	0
1	1	1	1	1	1	0	1	0
1	1	0	0	0	1	0	0	1
0	0	0	1	0	0	0	0	0
1	1	1	1	1	1	1	1	1
1	1	1	1	1	0	0	1	0
1	1	1	1	1	0	0	1	1
0	1	1	0	1	1	0	0	0
1	1	1	1	1	1	1	1	1
1	1	1	1	1	1	1	1	1
1	1	0	0	0	0	1	1	0
1	1	0	0	1	1	1	1	0
1	1	1	0	1	1	1	1	0
1	1	1	1	1	1	1	1	1
1	1	1	0	1	0	1	0	0
1	1	1	1	1	1	1	1	1
1	1	1	0	0	1	0	0	1

1	1	1	1	0	1	1	0	0
1	1	1	1	1	0	1	1	1
1	1	1	1	1	0	0	1	0
1	1	1	1	1	1	1	0	1
1	1	1	1	1	1	1	0	1
1	1	1	0	0	1	1	0	0
1	1	0	1	1	1	1	1	0
1	1	1	0	1	1	1	0	0
1	1	1	0	1	0	0	1	1
1	1	0	0	1	0	0	1	1
1	1	1	1	1	1	1	1	1
1	1	1	0	1	1	1	1	0
1	1	1	0	0	1	0	0	0
1	1	1	0	0	0	1	1	0
1	1	1	0	0	0	0	0	0
0	0	0	0	0	1	0	0	0
1	1	1	0	1	1	0	0	0
1	1	1	1	1	1	0	0	0
1	1	1	0	1	1	1	0	1
1	1	1	0	1	1	1	1	1
1	1	1	0	1	1	1	1	0
1	1	1	1	1	1	1	0	1
1	1	1	0	0	0	1	0	0
1	1	1	1	1	1	1	1	1
0	1	0	0	1	0	0	0	0
1	1	0	0	1	0	0	0	0
1	0	0	1	1	0	0	0	0
1	1	0	1	1	0	1	0	1
1	1	1	1	1	1	1	1	1
1	1	1	1	1	1	0	0	1
1	1	1	1	1	1	0	0	0
1	1	1	0	0	1	0	1	0
0	1	1	1	1	1	1	1	0
1	1	1	0	1	1	1	0	1
0	1	1	1	0	0	1	0	1
1	1	1	1	1	1	1	0	1
1	1	0	1	1	1	1	0	1
1	1	1	0	1	0	0	0	0
1	1	1	1	1	1	0	0	1
1	1	1	0	1	1	1	0	0
1	0	1	0	1	1	0	0	0
1	0	1	0	1	1	0	0	1
1	1	1	1	1	1	1	1	1
1	1	1	0	1	1	0	0	0
1	1	1	0	1	1	0	0	0
1	0	1	0	1	0	0	0	0
1	0	1	0	1	1	1	0	0
1	1	1	0	0	0	1	0	0
1	1	0	1	1	0	1	0	0
1	1	1	0	0	0	0	0	0

1	1	1	1	1	0	1	0	1
1	0	1	1	1	1	0	1	1
1	1	1	1	1	1	1	1	1
1	1	1	0	1	0	0	1	0
1	1	1	1	1	1	1	0	1
1	1	1	0	0	0	1	0	1
1	1	1	0	1	1	1	1	1
1	1	1	0	1	1	1	1	1
1	1	1	1	1	0	0	0	1
1	1	1	1	1	1	1	0	1
1	1	1	1	1	1	1	0	0
1	1	1	1	1	1	1	0	0
1	1	0	0	1	0	0	0	1
1	1	1	0	1	1	0	0	1
1	1	0	0	1	0	0	0	0
1	1	1	0	1	0	0	1	0
1	0	0	1	1	1	0	1	0
1	1	0	1	1	0	0	0	1
0	1	0	0	1	0	0	0	0
1	1	1	0	1	1	1	1	0
1	1	1	1	1	1	1	0	0
1	1	1	0	1	1	1	1	1
1	1	0	0	1	1	0	0	1
1	1	1	0	1	1	0	0	0
1	1	0	0	1	0	0	1	0
0	1	0	0	1	0	0	0	0
1	0	0	0	1	0	0	1	0
1	0	1	0	1	1	0	0	1
1	1	0	1	1	0	0	0	0
1	1	0	1	1	0	1	0	0
1	1	0	1	1	0	1	0	1
1	1	1	1	1	0	1	0	1
1	1	1	1	1	0	1	1	1
1	1	0	0	1	0	1	0	0
1	1	0	0	1	0	1	0	1
1	1	0	1	1	0	1	0	0
1	1	0	0	1	0	1	0	0
1	1	0	1	1	0	1	0	0
1	1	0	0	1	0	1	0	0
1	1	1	1	1	1	1	0	1
1	1	1	1	1	0	1	0	1
1	1	0	0	1	0	1	0	0
1	1	1	1	1	0	1	0	1
1	1	0	0	1	0	1	0	0
1	1	1	0	1	0	1	0	1
1	1	1	0	1	0	1	0	0
1	1	1	0	1	0	1	0	1
1	1	1	1	1	0	1	0	0
1	1	0	1	1	0	1	0	0
1	1	0	1	1	0	1	0	1

1	1	1	1	1	0	1	0	1
1	1	1	1	1	0	1	0	0
1	1	1	1	0	0	1	0	0
1	1	1	1	1	0	1	0	0
1	1	0	1	0	0	1	0	0
1	1	1	1	1	0	1	0	1
0	1	0	0	1	0	1	0	0
1	1	0	1	1	0	1	0	0
1	1	0	1	1	0	1	0	0
1	1	0	1	1	0	1	0	0
1	1	1	1	1	0	1	0	1
1	1	0	1	1	0	1	0	0
0	1	0	0	1	0	1	0	0
1	1	0	1	1	0	1	0	0
1	1	1	1	1	1	1	0	1
1	1	1	1	1	0	1	0	1
1	1	0	0	1	0	1	0	0
1	1	0	1	1	0	1	0	0
0	1	0	0	0	1	0	0	0
1	1	0	1	1	0	1	0	0
1	1	0	1	1	0	1	0	0
1	1	0	1	1	0	1	0	0
0	1	0	1	1	0	1	0	0
1	1	1	0	1	0	1	0	0
0	1	0	1	1	0	1	0	0
1	1	0	0	1	0	1	0	1
1	1	0	0	1	0	1	0	0
1	1	0	0	1	0	0	0	0
1	1	0	0	1	0	0	0	0
1	1	0	0	1	0	1	0	0
0	1	0	1	1	0	1	0	0
1	1	0	0	1	0	1	0	0
1	1	1	1	1	0	1	0	0
1	1	0	1	1	0	1	0	1
1	0	0	0	1	0	1	0	0
1	0	0	1	1	0	1	0	0
1	1	1	1	1	0	1	0	1
1	1	1	1	1	0	1	0	1
1	1	0	0	1	0	1	0	1
1	1	0	1	1	0	1	0	1
1	1	0	1	1	0	1	0	1
1	1	0	1	1	0	1	0	1
1	1	0	1	1	0	1	0	1
1	1	0	0	1	0	1	0	1
1	1	1	1	1	0	1	0	1
1	1	0	0	1	0	1	0	1
1	1	0	1	1	0	1	0	1
1	1	0	1	1	0	1	0	1
1	1	0	0	1	0	1	0	1
1	1	0	1	0	0	1	0	1

1	1	1	1	1	0	1	0	1
1	1	0	1	1	0	1	0	1
1	1	0	1	1	0	1	0	1

behavior_1	behavior_t	MNA_1	MNA_2	MNA_3	MNA_4	MNA_5	MNA_6	MNA_total
1	9	2	3	2	2	2	3	14
1	8	2	3	2	2	2	2	13
1	10	2	3	2	2	2	3	14
1	7	2	3	2	2	2	0	11
1	10	2	3	2	2	2	3	14
1	10	2	3	2	2	2	3	14
1	3	2	3	2	2	2	3	14
1	8	2	3	2	2	2	3	14
1	10	2	3	2	2	2	2	13
1	10	2	3	2	2	2	1	12
0	5	2	2	2	2	2	2	12
1	8	2	3	2	2	2	2	13
1	5	2	2	2	2	2	1	11
1	6	2	2	2	2	2	2	12
1	7	2	3	2	2	2	2	13
1	6	2	3	2	2	2	3	14
1	5	2	3	2	2	2	3	14
1	10	2	3	2	2	2	1	12
1	6	2	2	2	2	2	0	10
1	9	2	3	2	2	2	1	12
1	6	2	1	2	2	2	3	12
0	0	2	2	2	2	2	3	13
1	10	2	3	2	2	2	2	13
1	10	2	3	2	2	2	3	14
1	7	2	3	1	2	2	3	13
1	7	1	2	2	2	2	0	9
1	6	2	1	0	0	1	0	4
1	9	2	3	2	2	2	3	14
1	6	2	3	2	2	2	3	14
1	8	2	3	2	2	2	3	14
1	10	2	3	2	2	2	3	14
1	8	2	3	2	2	2	2	13
1	7	2	3	2	2	2	2	13
1	8	2	3	2	2	2	1	12
1	5	1	3	2	2	1	3	12
1	2	2	2	2	2	2	3	13
1	10	2	1	2	2	2	2	11
1	7	2	2	2	2	2	2	12
1	8	2	2	2	2	2	3	13
1	5	2	3	2	2	2	3	14
1	10	2	3	2	2	2	3	14
1	10	2	3	2	2	2	1	12
1	5	2	3	2	0	2	3	12
1	7	2	3	2	2	2	3	14
1	8	2	3	2	2	2	3	14
1	10	2	3	2	2	2	3	14
0	5	2	3	2	2	1	3	13
1	10	2	3	2	2	2	2	13
1	6	2	1	2	2	2	0	9

1	7	1	3	2	2	2	2	12
1	9	2	3	2	2	2	3	14
1	7	2	3	2	2	2	3	14
1	9	2	3	2	2	2	3	14
1	9	2	3	2	2	2	2	13
1	6	2	3	2	2	2	3	14
1	8	2	3	2	2	2	3	14
1	7	2	3	2	2	2	3	14
1	7	2	3	2	2	2	3	14
1	6	2	3	2	2	2	3	14
1	10	2	3	2	2	2	3	14
1	8	2	3	2	2	2	1	12
1	5	2	1	2	2	2	3	12
1	6	1	1	2	0	2	2	8
1	4	2	1	2	2	2	3	12
1	2	1	2	2	0	2	3	10
1	6	2	2	2	2	2	2	12
1	7	2	3	2	0	2	2	11
0	7	2	3	2	2	2	3	14
1	9	2	3	2	2	2	3	14
1	8	2	3	2	2	2	3	14
1	9	2	3	2	2	2	3	14
1	5	2	1	1	2	2	3	11
1	10	2	3	2	2	2	3	14
0	2	2	3	2	2	2	1	12
1	4	2	2	2	2	2	2	12
0	3	2	3	2	2	2	3	14
1	7	1	0	0	2	2	3	8
1	10	2	3	2	2	2	3	14
1	8	2	3	2	0	1	1	9
1	7	2	3	2	2	2	2	13
0	5	2	1	1	2	2	1	9
1	8	2	3	2	2	2	2	13
1	8	2	3	2	2	2	3	14
1	6	2	3	2	2	2	2	13
1	9	2	3	2	2	2	3	14
1	8	2	3	2	0	2	3	12
1	5	2	3	2	2	1	1	11
1	8	2	3	2	2	2	3	14
0	6	2	3	2	2	2	1	12
0	4	2	3	2	2	2	2	13
0	5	2	3	2	2	2	1	12
1	10	2	3	2	2	2	3	14
0	5	2	3	2	2	2	3	14
0	5	1	0	2	0	2	3	8
1	4	2	3	2	2	2	3	14
1	6	2	3	2	2	2	1	12
1	5	0	3	2	2	2	3	12
1	6	1	1	1	2	2	0	7
1	4	2	3	2	2	2	1	12

1	8	2	3	2	2	2	3	14
1	8	2	3	2	2	2	3	14
1	10	2	3	2	2	2	3	14
1	6	2	3	2	2	2	2	13
1	9	2	3	2	2	2	3	14
1	6	2	3	2	2	2	3	14
1	9	2	3	2	2	2	2	13
1	9	1	3	2	2	2	3	13
1	7	2	2	2	2	2	3	13
1	9	2	3	2	2	2	3	14
0	7	2	3	2	2	2	2	13
1	8	2	3	2	2	2	3	14
1	5	1	3	2	2	2	3	13
1	7	2	3	2	2	2	3	14
1	4	2	3	2	2	2	3	14
1	6	2	3	2	2	2	3	14
1	6	2	2	2	2	2	1	11
0	5	2	3	2	2	2	2	13
0	2	2	3	2	2	2	1	12
0	7	2	3	2	2	2	2	13
0	7	2	3	2	0	2	2	11
0	8	2	3	2	2	2	3	14
0	5	2	3	2	2	2	2	13
1	6	2	3	2	2	2	3	14
0	4	2	1	2	2	2	3	12
0	2	2	3	2	2	2	2	13
0	3	2	3	2	2	2	3	14
0	5	2	3	2	2	2	1	12
0	4	2	2	2	0	2	2	10
1	6	1	0	1	2	2	1	7
1	7	0	0	1	0	2	0	3
1	8	1	0	1	2	2	0	6
1	9	2	3	2	2	2	2	13
1	5	1	0	0	2	1	0	4
1	6	2	3	1	2	2	3	13
0	5	2	3	2	2	2	3	14
1	5	2	3	2	2	2	2	13
0	5	2	3	1	2	2	3	13
1	5	1	1	0	0	2	1	5
1	9	2	3	2	2	2	3	14
1	8	2	3	2	2	2	3	14
0	4	2	3	2	2	2	3	14
1	8	2	3	2	2	2	3	14
0	4	1	1	0	0	2	2	6
1	7	2	1	0	2	1	3	9
1	6	1	1	0	0	2	0	4
1	7	2	1	0	2	2	3	10
1	7	1	0	2	0	2	3	8
1	6	2	2	0	2	1	1	8
1	7	1	2	2	2	2	0	9

1	8	2	2	0	2	2	0	8
0	6	2	2	2	2	2	0	10
0	5	1	3	1	2	2	3	12
0	6	1	2	0	2	2	2	9
1	5	1	3	0	0	2	3	9
1	8	2	3	2	2	2	3	14
1	4	2	3	0	0	2	0	7
1	6	2	3	1	2	2	0	10
0	5	1	3	1	0	2	3	10
1	6	1	3	1	2	2	3	12
1	8	1	0	1	2	2	0	6
0	5	1	3	2	2	2	3	13
0	3	1	3	2	2	2	1	11
0	5	1	3	2	2	2	2	12
1	9	0	0	2	2	2	2	8
1	8	1	2	2	2	2	0	9
0	4	2	2	2	2	2	2	12
0	5	2	3	2	2	2	0	11
1	3	2	3	2	2	2	2	13
1	6	2	3	2	2	2	1	12
0	5	2	1	2	2	2	3	12
0	5	2	1	2	2	2	3	12
0	4	1	1	2	2	2	0	8
1	6	1	3	1	2	2	3	12
0	4	2	3	2	2	2	2	13
1	6	1	0	1	2	2	2	8
0	4	2	3	2	2	2	0	11
0	3	2	3	1	2	2	1	11
0	3	2	3	2	2	2	3	14
0	4	0	0	1	2	2	2	7
0	4	2	2	2	2	2	1	11
0	4	1	0	1	2	2	1	7
1	7	1	2	1	2	1	1	8
1	7	2	3	1	0	2	1	9
1	4	2	3	2	2	2	1	12
1	5	1	0	1	2	2	3	9
1	8	2	3	2	2	2	3	14
1	8	2	3	2	2	2	3	14
1	6	2	1	1	0	2	3	9
1	7	1	2	0	0	2	3	8
1	7	1	2	1	2	2	0	8
1	7	2	3	0	0	2	3	10
1	7	1	2	0	2	2	3	10
1	6	2	3	1	0	2	3	11
1	8	2	3	1	2	2	3	13
1	6	1	2	0	0	2	0	5
1	7	2	3	1	2	2	2	12
1	7	2	3	1	2	2	2	12
1	6	2	3	1	0	2	3	11
1	6	1	2	1	2	2	1	9

1	8	1	2	1	2	2	3	11
1	7	1	0	1	2	2	3	9
1	7	2	3	2	2	2	3	14

IADL_1	IADL_2	IADL_3	IADL_4	IADL_5	IADL_6	IADL_7	IADL_8	IADL_total
3	4	2	4	2	3	3	2	23
1	1	1	2	1	2	3	1	12
3	4	3	4	2	3	3	2	24
3	4	3	4	2	3	3	2	24
3	4	1	4	2	3	3	2	22
3	3	2	4	2	3	3	2	22
1	1	1	1	1	1	2	1	9
3	4	3	4	2	3	3	2	24
3	4	3	4	2	3	3	2	24
3	4	3	4	2	3	3	2	24
3	4	3	4	2	3	3	2	24
3	3	3	4	2	3	3	2	23
2	1	1	2	0	0	1	1	8
2	1	1	4	0	1	1	1	11
3	4	3	4	2	3	3	2	24
2	0	1	2	0	1	1	1	8
3	4	3	4	2	3	3	2	24
3	4	3	4	2	3	3	2	24
3	4	2	4	2	3	3	2	23
3	4	3	4	2	3	3	2	24
3	4	1	4	2	3	3	2	22
3	4	1	4	0	3	3	2	20
3	4	3	4	2	3	3	2	24
3	3	3	4	2	3	3	2	23
1	1	0	1	0	2	1	1	7
1	1	0	1	0	3	3	1	10
0	0	0	0	0	0	0	0	0
3	4	3	4	2	3	3	2	24
2	3	3	2	2	3	3	2	20
3	3	3	4	2	3	3	2	23
3	3	3	4	2	3	3	2	23
3	4	3	4	2	3	3	2	24
2	3	3	4	2	3	3	2	22
2	4	3	4	2	2	1	2	20
1	1	0	1	1	1	1	0	6
2	3	1	4	2	3	3	2	20
3	3	3	4	2	3	3	2	23
3	4	3	3	2	3	3	2	23
3	3	3	2	2	3	3	2	21
3	4	3	4	2	3	3	2	24
2	3	3	3	2	2	0	0	15
3	3	3	4	2	3	3	2	23
2	3	2	3	2	3	1	1	17
3	4	3	4	2	3	3	2	24
3	4	3	4	2	3	3	2	24
3	3	1	3	2	3	3	2	20
1	4	0	0	0	0	3	2	10
1	1	0	1	1	1	3	2	10
3	4	2	4	1	2	1	1	18

3	3	2	4	2	3	3	2	22
3	3	3	3	2	3	3	2	22
3	3	3	3	2	3	3	1	21
3	4	2	4	1	3	3	2	22
3	4	3	4	2	3	3	2	24
3	3	3	4	2	3	3	2	23
3	1	3	4	2	2	2	1	18
3	4	1	3	2	3	3	2	21
3	4	1	3	2	3	3	2	21
3	3	3	4	2	3	3	2	23
3	4	3	4	1	3	3	2	23
3	4	1	4	1	3	3	2	21
3	4	1	3	2	3	2	2	20
3	4	3	3	2	3	3	2	23
2	4	2	3	2	3	2	2	20
3	2	2	1	0	3	2	2	15
2	2	3	3	2	3	3	2	20
3	3	3	4	2	3	3	2	23
3	3	3	3	1	3	3	2	21
3	4	2	3	2	3	3	2	22
3	4	3	4	2	3	3	2	24
3	3	3	4	2	3	3	2	23
3	1	3	4	2	3	2	2	20
2	3	3	4	2	3	3	2	22
3	4	3	3	1	3	3	2	22
3	3	2	4	2	3	3	2	22
3	4	3	4	2	3	3	2	24
0	1	0	0	0	0	0	0	1
3	3	3	4	2	3	3	2	23
3	3	3	3	2	3	3	2	22
3	4	3	4	2	3	3	2	24
0	0	0	3	2	3	3	1	12
3	3	2	4	2	3	3	2	22
3	3	3	3	2	3	3	2	22
3	3	3	4	2	3	3	2	23
3	4	3	4	2	3	3	2	24
3	3	3	4	2	3	3	2	23
3	4	3	4	2	3	3	2	24
3	4	3	4	2	3	3	2	24
3	4	3	4	2	3	3	2	24
3	3	3	4	2	3	3	2	23
3	3	3	4	2	3	3	2	23
3	3	3	4	2	3	3	2	23
3	3	1	3	1	3	2	1	17
0	2	0	0	0	3	3	2	10
3	4	3	4	0	3	3	2	22
3	4	3	4	2	3	3	2	24
2	4	2	4	2	3	3	2	22
1	2	1	2	0	2	3	1	12
3	4	3	4	2	3	3	2	24

3	3	3	4	1	3	3	1	21
2	4	0	0	0	3	1	0	10
2	3	3	4	2	3	3	2	22
3	4	3	4	2	3	3	2	24
3	3	3	3	1	3	3	2	21
3	4	3	4	2	3	3	2	24
3	4	3	4	2	3	3	2	24
3	4	3	3	2	3	3	2	23
3	3	2	3	2	3	3	2	21
3	4	3	4	2	3	3	2	24
3	4	3	4	2	3	3	2	24
3	4	1	4	0	3	3	2	20
2	4	2	3	1	3	3	2	20
3	4	3	4	2	3	3	2	24
2	3	1	3	1	3	3	2	18
3	3	3	4	2	3	3	2	23
2	3	3	4	2	3	3	2	22
3	4	3	4	2	3	3	2	24
3	3	3	3	2	3	3	2	22
3	3	3	4	2	3	3	2	23
2	3	3	4	2	3	3	2	22
3	4	3	4	2	3	3	2	24
3	4	3	4	2	3	3	2	24
2	3	3	4	2	3	3	2	22
2	4	2	4	2	3	3	2	22
3	4	3	4	2	3	3	2	24
2	3	3	4	2	3	3	2	22
3	4	3	3	2	3	3	2	23
2	3	3	3	2	3	3	2	21
1	1	0	1	0	2	3	1	9
1	1	0	0	0	1	1	1	5
2	2	2	3	1	3	2	1	16
3	3	3	3	2	3	3	2	22
0	0	0	0	0	0	0	0	0
0	0	0	0	0	0	1	1	2
2	2	3	3	2	2	2	1	17
3	2	3	3	2	3	3	2	21
1	1	0	0	0	0	0	0	2
0	1	0	0	0	1	0	0	2
3	4	3	4	2	3	3	2	24
3	4	3	4	2	3	3	2	24
3	4	3	4	2	3	3	2	24
2	2	2	3	2	2	3	1	17
1	1	0	1	0	1	0	0	4
0	0	0	0	0	0	0	0	0
0	1	0	0	0	0	0	0	1
0	1	0	0	0	0	0	0	1
2	2	1	2	1	3	3	1	15
1	1	0	0	0	0	1	0	3
1	1	0	1	0	1	1	0	5

0	1	0	0	0	0	0	0	1
1	1	1	2	1	2	2	1	11
1	1	0	0	0	1	1	0	4
1	1	0	0	0	1	1	1	5
1	1	0	1	0	1	1	0	5
1	2	1	1	0	2	2	1	10
0	1	0	1	0	0	1	0	3
1	1	2	3	1	2	1	1	12
1	1	0	1	0	3	1	2	9
2	2	1	1	0	1	0	1	8
1	1	0	1	1	2	1	1	8
1	1	1	1	1	0	1	1	7
1	1	0	2	0	2	1	1	8
3	3	3	4	2	3	3	2	23
3	4	3	4	2	3	3	2	24
2	2	3	4	2	3	3	2	21
3	4	2	3	1	3	3	2	21
1	1	1	2	1	2	2	1	11
3	4	3	4	2	3	3	2	24
2	1	1	3	1	2	2	1	13
2	1	2	3	1	2	2	1	14
1	2	1	2	1	2	2	1	12
1	2	1	2	1	2	1	1	11
1	1	0	2	0	3	3	1	11
1	1	0	2	1	2	1	1	9
0	1	0	0	0	0	1	0	2
1	1	1	2	1	2	1	1	10
1	1	1	1	1	1	1	1	8
2	1	1	2	1	1	1	1	10
2	1	1	2	1	2	1	1	11
2	1	0	1	0	1	1	1	7
1	1	1	2	1	2	1	1	10
1	1	0	0	0	0	0	0	2
1	1	0	1	0	1	1	1	6
0	1	1	1	0	0	1	1	5
1	1	0	1	0	2	1	1	7
2	1	2	3	2	1	1	1	13
1	1	0	0	0	2	1	1	6
1	1	1	2	1	3	1	1	11
1	1	0	1	0	1	1	1	6
1	1	1	2	0	3	1	1	10
0	0	0	0	0	0	0	0	0
0	0	0	0	0	0	0	0	0
1	1	0	1	0	1	1	0	5
0	1	0	0	0	0	0	0	1
0	0	0	0	0	0	0	0	0
2	1	1	2	1	2	1	1	11
1	1	0	1	0	0	1	1	5
1	1	1	1	0	2	3	1	10
1	1	0	0	0	2	1	0	5

0	1	0	0	0	1	1	0	3
1	1	0	1	0	3	3	2	11
1	1	0	0	0	1	0	1	4

quality_1	quality_2	quality_3	quality_4	quality_5	quality_6	quality_7	quality_8	quality_9
1	1	1	2	2	2	2	2	2
2	2	2	2	2	2	2	2	1
0	0	2	0	1	0	0	0	0
0	1	0	2	2	1	1	0	0
0	0	0	0	1	0	0	0	0
0	0	0	0	0	0	0	0	0
2	0	1	0	0	0	0	0	0
0	0	0	0	0	0	0	0	0
0	1	1	1	1	1	0	2	0
0	0	0	0	0	0	0	0	0
0	0	0	2	2	2	0	1	0
0	0	1	0	0	0	0	0	0
0	0	0	0	0	0	0	0	0
3	2	1	0	0	0	0	1	1
0	0	0	0	0	0	0	0	0
1	0	0	0	1	0	0	0	0
0	0	0	0	0	0	0	0	0
0	0	0	0	0	0	0	0	0
1	2	2	2	2	0	1	1	0
1	1	1	1	1	1	1	1	1
1	0	0	0	1	0	0	0	0
0	0	2	2	3	2	0	0	0
0	0	0	0	0	0	0	0	0
0	0	0	0	2	1	0	1	1
0	0	0	0	0	0	1	0	0
0	0	0	0	0	0	0	0	0
1	1	1	2	1	1	1	1	1
1	0	2	2	2	2	2	0	1
0	0	0	2	2	3	2	1	2
0	0	0	2	1	2	1	1	1
0	0	2	2	2	2	1	0	2
2	1	2	3	3	3	3	1	2
1	0	0	1	1	0	0	0	0
1	1	0	1	1	1	1	1	1
1	0	0	1	1	1	0	0	1
2	2	2	3	2	2	2	2	1
1	0	1	1	2	1	0	0	0
2	0	1	2	1	1	1	0	1
2	2	2	2	1	1	1	2	2
0	0	0	0	0	0	0	0	0
2	1	0	0	0	0	1	2	2
0	0	0	0	0	0	0	0	0
1	0	0	0	2	1	2	0	1
0	0	0	2	2	2	3	0	0
0	0	1	0	1	0	0	0	0
0	0	0	0	0	0	0	0	0
2	1	2	1	2	2	2	1	2
1	1	2	1	2	2	2	1	2
1	1	2	1	1	1	2	1	1

3	3	2	2	2	2	2	1	3
0	0	0	0	0	0	1	0	0
2	2	1	2	1	1	2	1	1
0	0	0	0	0	0	0	0	0
0	0	0	0	1	1	0	0	0
1	1	1	2	2	2	2	1	2
1	0	0	1	0	0	2	0	1
0	0	0	0	0	0	0	0	0
2	1	2	2	2	2	1	1	2
2	1	1	2	2	2	2	1	1
1	1	2	2	1	1	2	0	1
0	0	0	1	1	0	2	0	1
1	1	1	2	1	1	1	0	1
2	1	2	1	2	1	1	1	1
0	1	1	1	1	2	1	1	1
1	1	0	2	2	1	2	1	1
1	0	0	0	2	1	0	0	0
2	1	0	0	0	3	0	0	0
1	0	3	2	2	2	1	0	2
2	1	2	1	1	2	2	2	1
1	1	1	1	1	1	1	1	1
0	0	0	0	0	0	0	0	0
0	1	0	1	2	0	1	1	0
0	2	0	0	2	0	0	0	0
0	1	0	0	0	0	1	0	0
0	0	0	0	0	0	0	0	0
0	0	0	0	1	1	0	0	0
0	0	0	0	0	0	0	0	0
0	0	0	0	0	0	0	0	0
0	0	0	0	0	0	0	0	0
0	0	0	2	2	2	0	0	1
0	0	0	0	1	0	0	0	0
1	1	1	0	1	2	1	0	0
0	0	0	0	0	1	0	0	0
0	0	0	0	0	0	0	1	0
0	0	0	0	0	0	0	0	0
2	2	2	2	2	2	1	0	2
0	0	0	0	1	0	0	0	0
0	0	0	0	2	1	0	0	0
0	0	0	0	0	0	0	0	0
1	0	0	0	0	1	0	0	0
0	0	0	0	0	0	0	0	0
1	1	1	2	1	1	1	0	1
1	1	0	2	2	2	2	2	3
2	3	0	3	3	2	3	2	2
0	0	0	1	3	3	2	1	1
0	0	0	1	1	1	0	0	0
1	1	0	0	0	0	1	0	0
0	0	0	0	0	0	0	0	0
2	0	1	1	1	1	1	0	0

0	0	0	0	0	0	0	0	0
0	0	2	2	2	1	1	1	1
2	0	0	1	1	1	2	0	0
1	0	0	0	0	0	2	1	0
0	0	2	2	3	3	1	0	1
0	0	0	1	1	2	1	1	1
1	1	0	0	0	0	0	0	0
0	0	0	1	1	1	0	0	0
0	0	0	2	1	1	0	0	0
0	0	0	0	0	0	0	0	0
0	0	0	0	0	0	0	0	0
0	0	0	0	0	0	0	0	0
1	1	1	1	1	1	2	1	1
0	0	0	2	2	1	2	2	2
1	1	1	1	1	1	1	1	1
0	0	0	2	0	0	0	1	0
0	0	0	0	0	1	0	0	0
0	0	0	0	1	1	0	1	0
0	0	1	0	0	1	1	0	0
2	0	0	0	3	1	0	1	0
0	0	0	0	0	0	0	0	0
0	0	0	0	1	1	0	0	0
0	0	0	0	2	2	0	0	0
0	0	0	0	2	3	0	0	1
0	0	0	1	0	0	0	0	0
0	2	0	1	0	1	0	0	0
0	0	0	1	1	0	0	0	1
0	0	0	0	0	1	0	0	0
0	2	0	0	0	0	0	0	0
0	1	0	1	0	0	1	0	0
0	0	0	0	0	0	0	0	0
1	1	1	1	1	1	1	1	1
0	0	0	0	0	0	0	0	0
1	1	1	1	1	1	1	1	1
0	0	0	0	0	0	0	0	0
0	0	0	0	0	0	0	0	0
0	0	0	0	0	0	0	0	0
0	0	0	0	0	0	0	0	0
1	1	1	1	1	1	1	1	1
0	0	0	0	0	1	1	2	1
1	0	1	1	1	2	1	1	1
0	0	0	0	0	0	0	0	0
0	1	2	1	2	2	1	1	1
2	2	2	2	2	1	2	2	2
1	1	1	1	1	1	1	1	1
2	2	2	2	2	2	2	1	1
1	1	1	1	1	1	1	1	1
0	0	0	0	0	0	0	0	0
1	1	1	1	1	1	1	1	1
1	1	1	1	1	1	1	1	1

0	0	0	0	0	0	0	0	0
0	0	0	0	0	0	0	0	0
1	1	0	1	0	0	0	0	0
0	0	0	0	0	0	0	0	0
0	0	0	0	0	0	0	0	0
1	1	1	1	1	1	1	1	1
0	2	1	2	2	2	2	1	1
1	0	0	0	0	0	0	0	0
1	1	1	1	1	1	1	1	1
1	1	0	0	0	0	0	0	0
0	0	0	0	0	0	0	0	0
0	0	0	0	0	0	0	0	0
0	0	0	0	0	0	0	0	0
0	0	0	0	0	0	0	0	0
0	0	0	0	0	0	0	0	0
0	0	0	0	0	0	0	0	0
0	0	0	0	0	0	0	0	0
0	0	0	0	0	0	0	0	0
0	0	0	0	0	0	0	0	0
0	0	0	0	0	0	0	0	0
1	1	1	1	1	1	1	1	1
1	1	1	1	1	1	1	1	1
1	2	1	1	1	1	1	1	1
1	1	1	1	1	1	1	1	1
0	0	0	0	0	0	0	0	0
1	1	1	1	1	1	1	1	1
1	1	1	1	1	1	1	1	1
1	1	1	1	1	1	1	1	1
0	0	0	0	0	0	0	0	0
1	1	1	1	1	1	1	1	1
0	0	0	0	0	0	0	0	0
0	0	0	0	0	0	0	0	0
0	0	0	0	0	0	0	0	0
1	1	1	2	1	1	1	1	0
0	0	0	0	0	0	0	0	0
0	0	0	0	0	0	0	0	0
0	0	0	0	0	0	0	0	0
0	0	0	0	0	0	0	0	0
1	1	1	1	1	1	1	1	1
1	1	1	1	1	1	2	0	0
1	1	1	1	1	1	1	1	1
0	0	0	0	0	0	0	0	0
0	0	0	0	0	0	0	0	0
0	0	0	0	0	0	0	0	0
0	0	0	0	0	0	0	0	0
0	0	0	0	0	0	0	0	0
0	0	0	0	0	0	0	0	0
0	0	0	0	0	0	0	0	0
0	0	0	0	0	0	0	0	0
0	0	0	0	0	0	0	0	0

0	0	0	0	0	0	0	0	0
0	0	0	0	0	0	0	0	0
0	0	0	0	0	0	0	0	0

quality_10	quality_11	quality_12	quality_13	quality_14	quality_tot	quality_fur	quality_phy	quality_psy
2	2	2	2	2	25	1.00	1.50	2.00
2	1	0	1	0	21	2.00	2.00	2.00
0	0	0	0	0	3	0.00	1.00	0.50
0	0	0	1	0	8	0.50	1.00	1.50
0	0	0	0	0	1	0.00	0.00	0.50
0	0	0	0	0	0	0.00	0.00	0.00
0	0	0	0	0	3	1.00	0.50	0.00
0	0	0	0	0	0	0.00	0.00	0.00
0	0	0	0	0	7	0.50	1.00	1.00
0	0	0	0	0	0	0.00	0.00	0.00
0	0	1	1	1	10	0.00	1.00	2.00
0	0	0	1	0	2	0.00	0.50	0.00
0	0	0	0	0	0	0.00	0.00	0.00
1	1	1	1	1	13	2.50	0.50	0.00
0	0	0	0	0	0	0.00	0.00	0.00
0	0	0	0	0	2	0.50	0.00	0.50
0	0	0	0	0	0	0.00	0.00	0.00
0	0	0	0	0	0	0.00	0.00	0.00
0	0	0	2	0	13	1.50	2.00	1.00
1	1	1	1	1	14	1.00	1.00	1.00
0	0	0	0	0	2	0.50	0.00	0.50
0	0	0	1	0	10	0.00	2.00	2.50
0	0	0	0	0	0	0.00	0.00	0.00
0	0	0	0	0	5	0.00	0.00	1.50
0	0	0	1	0	2	0.00	0.00	0.00
0	0	0	0	0	0	0.00	0.00	0.00
1	1	1	1	1	15	1.00	1.50	1.00
1	1	0	1	1	16	0.50	2.00	2.00
2	2	2	1	0	19	0.00	1.00	2.50
0	1	0	1	1	11	0.00	1.00	1.50
2	1	0	0	0	14	0.00	2.00	2.00
3	2	2	2	2	31	1.50	2.50	3.00
1	0	0	0	0	4	0.50	0.50	0.50
1	1	0	0	0	10	1.00	0.50	1.00
0	1	1	1	0	8	0.50	0.50	1.00
3	2	1	2	2	28	2.00	2.50	2.00
0	0	0	1	0	7	0.50	1.00	1.50
2	1	1	1	1	15	1.00	1.50	1.00
2	1	1	3	2	24	2.00	2.00	1.00
0	0	0	0	0	0	0.00	0.00	0.00
1	2	1	2	1	15	1.50	0.00	0.00
0	0	0	0	0	0	0.00	0.00	0.00
1	0	0	2	0	10	0.50	0.00	1.50
3	0	0	1	0	13	0.00	1.00	2.00
0	0	0	0	0	2	0.00	0.50	0.50
2	0	0	2	2	6	0.00	0.00	0.00
0	1	1	0	0	17	1.50	1.50	2.00
1	2	1	2	1	21	1.00	1.50	2.00
0	1	1	2	1	16	1.00	1.50	1.00

1	1	2	2	1	27	3.00	2.00	2.00
0	0	0	1	0	2	0.00	0.00	0.00
1	1	1	2	1	19	2.00	1.50	1.00
0	0	0	0	0	0	0.00	0.00	0.00
0	0	0	0	0	2	0.00	0.00	1.00
2	0	0	2	0	18	1.00	1.50	2.00
0	1	0	0	0	6	0.50	0.50	0.00
0	0	0	0	0	0	0.00	0.00	0.00
1	1	1	1	1	20	1.50	2.00	2.00
1	2	1	1	1	20	1.50	1.50	2.00
0	1	1	1	1	15	1.00	2.00	1.00
1	0	0	0	0	6	0.00	0.50	0.50
0	0	0	0	0	9	1.00	1.50	1.00
1	1	1	2	1	18	1.50	1.50	1.50
2	0	1	1	1	14	0.50	1.00	1.50
2	2	0	2	0	17	1.00	1.00	1.50
1	0	0	0	0	5	0.50	0.00	1.50
0	0	0	0	2	8	1.50	0.00	1.50
1	0	1	1	1	17	0.50	2.50	2.00
2	1	1	2	2	22	1.50	1.50	1.50
1	1	1	1	1	14	1.00	1.00	1.00
0	0	0	0	0	0	0.00	0.00	0.00
0	0	0	0	0	6	0.50	0.50	1.00
0	0	0	0	0	4	1.00	0.00	1.00
0	0	0	0	0	2	0.50	0.00	0.00
1	0	0	0	0	1	0.00	0.00	0.00
0	0	0	0	0	2	0.00	0.00	1.00
0	0	0	0	0	0	0.00	0.00	0.00
0	0	0	0	0	0	0.00	0.00	0.00
0	0	0	0	0	0	0.00	0.00	0.00
0	0	0	1	0	8	0.00	1.00	2.00
0	0	0	0	0	1	0.00	0.00	0.50
1	1	1	1	0	11	1.00	0.50	1.50
0	0	0	0	0	1	0.00	0.00	0.50
0	0	0	1	0	2	0.00	0.00	0.00
0	0	0	0	0	0	0.00	0.00	0.00
2	2	0	1	1	21	2.00	2.00	2.00
0	0	0	0	0	1	0.00	0.00	0.50
0	0	0	0	0	3	0.00	0.00	1.50
0	0	0	0	0	0	0.00	0.00	0.00
0	0	1	0	1	4	0.50	0.00	0.50
0	0	0	0	0	0	0.00	0.00	0.00
1	0	0	0	0	10	1.00	1.50	1.00
2	2	1	2	1	23	1.00	1.00	2.00
1	1	1	2	1	26	2.50	1.50	2.50
3	0	0	3	1	18	0.00	0.50	3.00
0	0	0	0	0	3	0.00	0.50	1.00
0	0	0	1	1	5	1.00	0.00	0.00
0	0	0	0	0	0	0.00	0.00	0.00
0	0	0	0	0	7	1.00	1.00	1.00

0	0	0	0	0	0	0.00	0.00	0.00
2	1	1	0	1	15	0.00	2.00	1.50
1	1	1	0	1	11	1.00	0.50	1.00
0	0	0	0	0	4	0.50	0.00	0.00
0	0	0	1	0	13	0.00	2.00	3.00
0	1	1	1	1	11	0.00	0.50	1.50
0	0	0	0	0	2	1.00	0.00	0.00
0	0	0	0	0	3	0.00	0.50	1.00
0	0	0	0	0	4	0.00	1.00	1.00
0	0	0	0	0	0	0.00	0.00	0.00
0	0	0	0	0	0	0.00	0.00	0.00
0	0	0	0	0	0	0.00	0.00	0.00
1	1	1	1	1	15	1.00	1.00	1.00
1	1	0	2	1	16	0.00	1.00	1.50
1	1	1	1	1	14	1.00	1.00	1.00
0	0	0	0	0	3	0.00	1.00	0.00
0	0	0	0	0	1	0.00	0.00	0.50
0	0	0	0	0	3	0.00	0.00	1.00
0	0	0	0	0	3	0.00	0.50	0.50
0	0	0	0	0	7	1.00	0.00	2.00
0	1	0	0	2	3	0.00	0.00	0.00
0	0	0	0	0	2	0.00	0.00	1.00
1	0	0	0	0	5	0.00	0.00	2.00
0	0	0	0	0	6	0.00	0.00	2.50
0	0	0	0	0	1	0.00	0.50	0.00
0	0	0	0	0	4	1.00	0.50	0.50
0	0	0	0	0	3	0.00	0.50	0.50
1	0	0	0	0	2	0.00	0.00	0.50
0	0	0	0	0	2	1.00	0.00	0.00
0	0	0	1	0	4	0.50	0.50	0.00
0	0	0	0	0	0	0.00	0.00	0.00
1	1	1	1	1	14	1.00	1.00	1.00
0	0	0	0	0	0	0.00	0.00	0.00
1	1	1	1	1	14	1.00	1.00	1.00
0	0	0	0	0	0	0.00	0.00	0.00
0	0	0	0	0	0	0.00	0.00	0.00
0	0	0	0	0	0	0.00	0.00	0.00
0	0	0	0	0	0	0.00	0.00	0.00
1	1	1	1	1	14	1.00	1.00	1.00
0	0	0	0	1	6	0.00	0.00	0.50
0	0	0	1	1	11	0.50	1.00	1.50
0	0	0	0	0	0	0.00	0.00	0.00
1	1	1	1	1	16	0.50	1.50	2.00
2	2	2	2	2	27	2.00	2.00	1.50
1	1	1	1	1	14	1.00	1.00	1.00
1	1	1	1	1	21	2.00	2.00	2.00
1	1	1	1	1	14	1.00	1.00	1.00
0	0	0	0	0	0	0.00	0.00	0.00
1	1	1	1	1	14	1.00	1.00	1.00
1	1	1	1	1	14	1.00	1.00	1.00

0	0	0	0	0	0	0.00	0.00	0.00
0	0	0	0	0	0	0.00	0.00	0.00
0	0	0	0	0	3	1.00	0.50	0.00
0	0	0	0	0	0	0.00	0.00	0.00
0	0	0	0	0	0	0.00	0.00	0.00
1	1	1	1	1	14	1.00	1.00	1.00
1	1	2	2	2	21	1.00	1.50	2.00
0	0	0	0	0	1	0.50	0.00	0.00
1	1	0	1	1	13	1.00	1.00	1.00
0	0	0	0	0	2	1.00	0.00	0.00
0	0	0	0	0	0	0.00	0.00	0.00
0	0	0	0	0	0	0.00	0.00	0.00
0	0	0	0	0	0	0.00	0.00	0.00
0	0	0	0	0	0	0.00	0.00	0.00
0	0	0	0	0	0	0.00	0.00	0.00
0	0	0	0	0	0	0.00	0.00	0.00
0	0	0	0	0	0	0.00	0.00	0.00
0	0	0	0	0	0	0.00	0.00	0.00
0	0	0	0	0	0	0.00	0.00	0.00
0	0	0	0	0	0	0.00	0.00	0.00
1	1	1	1	1	14	1.00	1.00	1.00
1	1	1	1	1	14	1.00	1.00	1.00
1	1	1	1	1	15	1.50	1.00	1.00
0	0	0	0	0	9	1.00	1.00	1.00
0	0	0	0	0	0	0.00	0.00	0.00
1	1	1	1	1	14	1.00	1.00	1.00
1	1	1	1	1	14	1.00	1.00	1.00
1	1	1	1	1	14	1.00	1.00	1.00
0	0	0	0	0	0	0.00	0.00	0.00
1	1	1	1	1	14	1.00	1.00	1.00
0	0	0	0	0	0	0.00	0.00	0.00
0	0	0	0	0	0	0.00	0.00	0.00
0	0	0	0	0	0	0.00	0.00	0.00
0	0	0	1	1	11	1.00	1.50	1.00
0	0	0	0	0	0	0.00	0.00	0.00
0	0	0	0	0	0	0.00	0.00	0.00
0	0	0	0	0	0	0.00	0.00	0.00
0	0	0	0	0	0	0.00	0.00	0.00
1	0	0	1	1	12	1.00	1.00	1.00
0	0	0	1	1	10	1.00	1.00	1.00
1	1	1	1	1	14	1.00	1.00	1.00
0	0	0	0	0	0	0.00	0.00	0.00
0	0	0	0	0	0	0.00	0.00	0.00
0	0	0	0	0	0	0.00	0.00	0.00
0	0	0	0	0	0	0.00	0.00	0.00
0	0	0	0	0	0	0.00	0.00	0.00
0	0	0	0	0	0	0.00	0.00	0.00
0	0	0	0	0	0	0.00	0.00	0.00
0	0	0	0	0	0	0.00	0.00	0.00
0	0	0	0	0	0	0.00	0.00	0.00

0	0	0	0	0	0	0.00	0.00	0.00
0	0	0	0	0	0	0.00	0.00	0.00
0	0	0	0	0	0	0.00	0.00	0.00

quality_Phy	quality_psy	quality_Soc	quality_obs	OHL_1	OHL_2_1	OHL_2_2	OHL_2_3	OHL_3
2.00	2.00	2.00	2.00	0	1	0	1	0
2.00	1.50	0.50	0.50	0	1	0	0	0
0.00	0.00	0.00	0.00	0	1	0	1	1
0.50	0.00	0.00	0.50	1	1	1	1	1
0.00	0.00	0.00	0.00	1	1	0	1	1
0.00	0.00	0.00	0.00	1	1	0	1	1
0.00	0.00	0.00	0.00	0	1	1	1	0
0.00	0.00	0.00	0.00	1	1	1	0	1
1.00	0.00	0.00	0.00	1	1	0	1	1
0.00	0.00	0.00	0.00	1	1	1	1	1
0.50	0.00	0.50	1.00	1	1	1	1	1
0.00	0.00	0.00	0.50	1	1	1	1	1
0.00	0.00	0.00	0.00	1	1	1	1	1
0.50	1.00	1.00	1.00	0	1	1	1	0
0.00	0.00	0.00	0.00	0	1	0	0	0
0.00	0.00	0.00	0.00	1	1	0	1	1
0.00	0.00	0.00	0.00	1	1	1	1	1
0.00	0.00	0.00	0.00	1	1	1	1	1
1.00	0.00	0.00	1.00	0	1	0	1	1
1.00	1.00	1.00	1.00	0	1	0	1	0
0.00	0.00	0.00	0.00	0	1	1	1	1
0.00	0.00	0.00	0.50	0	1	1	1	1
0.00	0.00	0.00	0.00	0	1	1	1	1
0.50	0.50	0.00	0.00	0	1	1	1	1
0.50	0.00	0.00	0.50	1	1	1	1	0
0.00	0.00	0.00	0.00	1	1	0	1	0
1.00	1.00	1.00	1.00	0	1	1	1	0
1.00	1.00	0.50	1.00	1	1	0	1	1
1.50	2.00	2.00	0.50	0	1	0	1	0
1.00	0.50	0.50	1.00	1	1	1	1	1
0.50	2.00	0.50	0.00	0	1	0	1	1
2.00	2.50	2.00	2.00	0	1	1	1	0
0.00	0.50	0.00	0.00	0	1	0	0	0
1.00	1.00	0.50	0.00	0	1	0	0	1
0.00	0.50	1.00	0.50	0	1	1	0	1
2.00	2.00	1.50	2.00	1	0	0	1	0
0.00	0.00	0.00	0.50	0	1	0	1	1
0.50	1.50	1.00	1.00	1	1	0	1	0
1.50	2.00	1.00	2.50	1	1	0	1	0
0.00	0.00	0.00	0.00	1	1	1	1	1
1.50	1.50	1.50	1.50	1	0	0	1	0
0.00	0.00	0.00	0.00	0	1	1	1	0
1.00	1.00	0.00	1.00	0	1	1	0	0
1.50	1.50	0.00	0.50	0	1	1	1	1
0.00	0.00	0.00	0.00	1	1	1	1	1
0.00	1.00	0.00	2.00	0	0	1	1	0
1.50	1.00	1.00	0.00	0	1	1	1	0
1.50	1.50	1.50	1.50	0	0	1	1	1
1.50	0.50	1.00	1.50	0	0	0	1	0

1.50	2.00	1.50	1.50	1	1	0	1	0
0.50	0.00	0.00	0.50	0	1	1	1	1
1.50	1.00	1.00	1.50	1	1	0	1	1
0.00	0.00	0.00	0.00	0	1	1	1	1
0.00	0.00	0.00	0.00	1	1	0	1	1
1.50	2.00	0.00	1.00	1	1	1	1	1
1.00	0.50	0.50	0.00	0	0	1	1	0
0.00	0.00	0.00	0.00	0	1	0	1	1
1.00	1.50	1.00	1.00	0	1	0	1	1
1.50	1.00	1.50	1.00	0	1	0	1	1
1.00	0.50	1.00	1.00	0	1	1	1	0
1.00	1.00	0.00	0.00	0	1	0	1	0
0.50	0.50	0.00	0.00	1	1	1	1	1
1.00	1.00	1.00	1.50	0	0	0	0	0
1.00	1.50	0.50	1.00	1	0	0	1	1
1.50	1.50	1.00	1.00	1	1	1	1	1
0.00	0.50	0.00	0.00	0	1	1	1	1
0.00	0.00	0.00	1.00	0	1	1	1	1
0.50	1.50	0.50	1.00	0	1	0	1	1
2.00	1.50	1.00	2.00	1	1	1	1	1
1.00	1.00	1.00	1.00	0	1	1	1	1
0.00	0.00	0.00	0.00	0	1	1	1	1
1.00	0.00	0.00	0.00	0	0	1	0	0
0.00	0.00	0.00	0.00	0	1	0	1	0
0.50	0.00	0.00	0.00	0	0	1	1	1
0.00	0.50	0.00	0.00	0	0	0	1	0
0.00	0.00	0.00	0.00	0	0	0	1	1
0.00	0.00	0.00	0.00	0	0	0	1	0
0.00	0.00	0.00	0.00	0	1	1	1	1
0.00	0.00	0.00	0.00	1	1	1	1	1
0.00	0.50	0.00	0.50	1	1	1	1	1
0.00	0.00	0.00	0.00	1	1	1	1	1
0.50	0.50	1.00	0.50	0	1	0	1	0
0.00	0.00	0.00	0.00	0	0	1	1	1
0.50	0.00	0.00	0.50	0	0	0	0	0
0.00	0.00	0.00	0.00	0	1	0	1	0
0.50	2.00	1.00	1.00	0	0	0	1	0
0.00	0.00	0.00	0.00	0	0	1	1	0
0.00	0.00	0.00	0.00	1	1	1	1	0
0.00	0.00	0.00	0.00	0	0	1	1	0
0.00	0.00	0.50	0.50	0	0	0	1	0
0.00	0.00	0.00	0.00	1	1	0	1	1
0.50	1.00	0.00	0.00	0	1	0	1	1
2.00	2.50	1.50	1.50	0	1	1	1	1
2.50	1.50	1.00	1.50	1	1	1	1	1
1.50	2.00	0.00	2.00	0	0	0	1	1
0.00	0.00	0.00	0.00	1	1	0	1	1
0.50	0.00	0.00	1.00	0	0	0	1	0
0.00	0.00	0.00	0.00	1	1	1	1	0
0.50	0.00	0.00	0.00	0	1	0	1	1

0.00	0.00	0.00	0.00	0	1	1	1	1
1.00	1.50	1.00	0.50	0	1	0	1	1
1.00	0.50	1.00	0.50	0	1	0	1	1
1.50	0.00	0.00	0.00	0	0	1	1	0
0.50	0.50	0.00	0.50	0	1	0	1	1
1.00	0.50	1.00	1.00	1	1	0	1	1
0.00	0.00	0.00	0.00	0	0	0	1	0
0.00	0.00	0.00	0.00	0	0	0	1	0
0.00	0.00	0.00	0.00	0	1	1	0	1
0.00	0.00	0.00	0.00	0	1	1	1	0
0.00	0.00	0.00	0.00	0	1	1	1	1
0.00	0.00	0.00	0.00	0	1	1	1	1
1.50	1.00	1.00	1.00	0	1	1	1	1
2.00	1.50	0.50	1.50	0	1	1	1	1
1.00	1.00	1.00	1.00	0	1	1	1	1
0.50	0.00	0.00	0.00	0	0	0	0	0
0.00	0.00	0.00	0.00	0	1	0	1	1
0.50	0.00	0.00	0.00	0	0	0	1	1
0.50	0.00	0.00	0.00	0	0	1	1	1
0.50	0.00	0.00	0.00	1	1	1	1	1
0.00	0.00	0.50	1.00	0	0	1	1	1
0.00	0.00	0.00	0.00	0	0	0	1	0
0.00	0.50	0.00	0.00	0	1	1	1	1
0.00	0.50	0.00	0.00	0	0	0	1	1
0.00	0.00	0.00	0.00	0	0	0	1	0
0.00	0.00	0.00	0.00	0	0	1	1	0
0.00	0.50	0.00	0.00	0	0	1	1	0
0.00	0.50	0.00	0.00	0	0	1	1	1
0.00	0.00	0.00	0.00	0	1	0	1	1
0.50	0.00	0.00	0.50	0	1	1	1	0
0.00	0.00	0.00	0.00	0	1	1	1	0
1.00	1.00	1.00	1.00	1	1	0	1	0
0.00	0.00	0.00	0.00	1	1	1	1	0
1.00	1.00	1.00	1.00	0	0	0	1	0
0.00	0.00	0.00	0.00	0	0	0	1	0
0.00	0.00	0.00	0.00	0	0	0	1	0
0.00	0.00	0.00	0.00	1	1	1	1	0
0.00	0.00	0.00	0.00	0	1	1	1	0
1.00	1.00	1.00	1.00	1	1	1	1	0
1.50	0.50	0.00	0.50	0	1	0	1	1
1.00	0.50	0.00	1.00	0	1	1	1	1
0.00	0.00	0.00	0.00	1	1	1	1	1
1.00	1.00	1.00	1.00	0	0	1	1	0
2.00	2.00	2.00	2.00	0	0	0	1	0
1.00	1.00	1.00	1.00	0	0	0	1	0
1.50	1.00	1.00	1.00	0	0	0	1	0
1.00	1.00	1.00	1.00	1	1	1	1	0
0.00	0.00	0.00	0.00	0	0	1	1	0
1.00	1.00	1.00	1.00	0	0	0	0	0
1.00	1.00	1.00	1.00	0	1	0	1	0

0.00	0.00	0.00	0.00	0	0	0	1	0
0.00	0.00	0.00	0.00	0	1	0	1	0
0.00	0.00	0.00	0.00	0	1	0	1	0
0.00	0.00	0.00	0.00	0	0	0	1	0
0.00	0.00	0.00	0.00	0	0	0	1	0
1.00	1.00	1.00	1.00	0	1	1	1	0
1.50	1.00	1.50	2.00	1	1	1	1	0
0.00	0.00	0.00	0.00	0	1	1	1	0
1.00	1.00	0.50	1.00	1	1	1	1	0
0.00	0.00	0.00	0.00	0	1	0	1	0
0.00	0.00	0.00	0.00	1	1	1	1	0
0.00	0.00	0.00	0.00	0	1	0	1	0
0.00	0.00	0.00	0.00	0	1	0	1	0
0.00	0.00	0.00	0.00	1	1	1	1	0
0.00	0.00	0.00	0.00	1	1	1	1	0
0.00	0.00	0.00	0.00	1	1	1	1	0
0.00	0.00	0.00	0.00	1	1	0	1	0
0.00	0.00	0.00	0.00	1	1	1	1	0
0.00	0.00	0.00	0.00	0	1	1	1	0
0.00	0.00	0.00	0.00	0	1	0	1	0
1.00	1.00	1.00	1.00	0	1	1	1	0
1.00	1.00	1.00	1.00	1	0	0	1	0
1.00	1.00	1.00	1.00	0	1	0	1	0
1.00	0.50	0.00	0.00	0	1	1	1	0
0.00	0.00	0.00	0.00	0	1	1	1	0
1.00	1.00	1.00	1.00	0	1	0	1	0
1.00	1.00	1.00	1.00	0	1	0	1	0
1.00	1.00	1.00	1.00	0	1	0	1	0
0.00	0.00	0.00	0.00	0	1	1	1	0
1.00	1.00	1.00	1.00	0	1	0	1	0
0.00	0.00	0.00	0.00	0	0	0	1	0
0.00	0.00	0.00	0.00	1	1	1	1	0
0.00	0.00	0.00	0.00	0	0	0	0	0
1.00	0.00	0.00	1.00	1	1	1	1	0
0.00	0.00	0.00	0.00	1	1	0	1	0
0.00	0.00	0.00	0.00	1	1	1	1	0
0.00	0.00	0.00	0.00	0	1	0	1	0
0.00	0.00	0.00	0.00	1	1	1	1	0
1.00	1.00	0.00	1.00	1	1	1	1	0
1.00	0.00	0.00	1.00	1	1	1	1	0
1.00	1.00	1.00	1.00	1	1	1	1	0
0.00	0.00	0.00	0.00	0	1	0	1	0
0.00	0.00	0.00	0.00	0	1	1	1	0
0.00	0.00	0.00	0.00	0	1	1	1	0
0.00	0.00	0.00	0.00	1	1	1	1	0
0.00	0.00	0.00	0.00	0	0	0	1	0
0.00	0.00	0.00	0.00	0	0	0	1	0
0.00	0.00	0.00	0.00	0	0	1	1	0
0.00	0.00	0.00	0.00	0	1	0	1	0
0.00	0.00	0.00	0.00	0	1	1	1	0

0.00	0.00	0.00	0.00	1	1	1	1	0
0.00	0.00	0.00	0.00	1	1	1	1	0
0.00	0.00	0.00	0.00	1	1	1	1	0

OHL_4	OHL_5	OHL_6	OHL_7	OHL_8	OHL_9	OHL_10	OHL_11	OHL_12
0	1	0	1	1	0	0	0	1
0	0	0	1	0	1	1	1	1
0	0	1	1	0	1	1	1	1
1	1	1	1	1	0	0	1	1
0	0	0	1	0	1	1	1	1
0	0	0	1	0	1	1	1	1
0	0	1	1	0	1	1	1	1
0	1	0	1	1	0	1	1	1
0	1	0	1	1	0	1	1	1
0	1	1	1	1	0	1	1	1
0	1	0	1	1	0	1	1	1
0	1	1	1	1	0	1	1	1
0	1	1	1	1	0	0	1	1
0	1	1	1	1	1	1	1	1
0	0	0	1	1	0	1	0	1
0	1	1	1	0	1	1	1	1
0	1	0	1	1	0	0	1	1
0	1	1	1	1	0	0	1	0
0	1	0	1	0	0	0	1	1
0	0	0	0	0	0	1	1	1
0	1	1	1	1	1	1	1	1
0	1	1	1	1	1	1	1	1
1	1	1	1	1	1	1	1	1
1	1	1	1	1	1	1	1	1
0	0	0	0	1	1	0	1	1
0	1	0	0	1	1	0	1	1
0	0	0	1	0	0	0	0	1
0	1	1	1	1	1	1	1	1
0	0	1	1	1	1	1	1	1
0	1	0	1	1	1	1	0	1
0	1	1	1	1	1	1	1	1
0	1	1	1	1	1	1	0	1
0	1	1	1	1	1	1	1	1
0	0	0	1	0	1	1	0	1
0	0	1	1	1	1	1	0	1
1	1	0	1	1	1	1	0	1
1	1	0	1	0	1	1	0	1
0	0	0	1	0	1	1	0	1
0	0	0	1	0	1	1	0	1
1	1	1	1	1	1	1	1	1
0	0	1	1	0	0	1	1	1
0	1	0	1	1	1	1	0	1
0	1	0	1	1	1	1	1	1
0	1	1	1	1	0	1	1	1
1	1	1	1	1	1	1	1	1
0	1	1	1	1	0	1	1	1
0	0	0	1	0	0	0	1	1
0	0	0	1	1	1	1	1	1
0	1	1	1	0	0	0	0	1

0	1	0	1	1	1	1	1	1
0	1	0	1	1	1	1	1	1
1	0	1	1	0	1	1	0	1
1	1	0	1	1	1	1	1	1
1	1	1	1	1	1	1	1	1
1	1	1	1	1	1	1	1	1
0	0	0	1	1	0	1	1	1
1	1	0	0	1	1	1	0	1
0	1	0	1	1	1	1	1	1
0	1	0	1	1	1	1	1	1
0	0	0	1	1	1	1	0	1
0	1	1	1	1	1	1	1	1
1	1	1	1	1	1	1	0	1
0	0	1	0	0	0	1	0	1
0	1	0	0	1	1	1	1	1
0	1	1	1	1	0	1	1	1
1	1	1	1	1	1	1	1	1
0	1	0	1	0	0	1	1	0
0	1	1	1	1	1	1	1	1
0	1	1	1	1	1	1	1	1
0	1	1	1	1	1	1	1	1
0	1	1	1	1	1	1	1	1
0	0	0	1	0	1	1	1	1
0	1	0	1	0	0	1	1	1
0	1	1	1	1	1	1	1	1
0	1	0	1	1	0	0	0	1
0	0	1	1	1	1	1	0	1
0	0	0	0	0	0	0	1	1
0	0	0	1	1	1	1	0	1
1	1	1	1	1	1	1	1	1
0	1	0	1	1	1	1	1	1
0	1	0	1	1	1	1	1	1
0	0	1	1	0	1	1	1	1
0	1	1	1	0	1	1	1	1
0	0	0	1	0	0	1	0	1
0	1	1	1	1	1	0	1	1
0	0	0	1	0	0	1	1	1
0	0	0	1	1	1	0	1	1
0	0	0	1	0	0	1	1	1
0	0	0	0	0	1	1	1	1
0	0	1	1	0	1	1	0	1
0	1	1	1	1	1	1	1	1
0	0	0	1	0	1	1	0	1
1	1	1	1	1	1	1	1	1
0	1	1	1	1	1	1	0	1
0	0	0	1	1	1	0	0	1
0	1	0	1	1	1	1	1	1
0	0	1	0	0	1	1	0	1
0	1	0	0	1	1	0	1	1
0	0	0	1	0	0	1	0	1

0	0	1	1	0	1	0	0	1
0	1	1	1	1	1	1	1	1
0	1	1	1	1	1	1	1	1
0	1	0	0	1	1	1	0	1
0	1	1	1	1	1	1	1	1
0	1	0	1	1	1	0	1	1
0	1	1	1	1	1	1	0	1
0	1	1	1	1	0	1	0	1
1	1	1	1	1	0	0	1	1
0	0	0	1	0	0	1	1	1
0	1	1	1	1	1	1	1	1
0	1	1	1	1	1	1	1	1
0	1	0	0	0	1	1	0	1
0	1	1	0	1	1	1	1	1
0	1	1	1	1	1	1	1	1
0	0	0	1	0	0	0	1	1
0	1	0	1	1	0	0	1	1
0	0	1	1	1	0	1	0	1
0	0	1	1	0	0	1	0	1
1	1	1	1	1	1	1	1	1
0	1	0	1	1	1	0	0	1
0	0	1	0	0	0	0	0	1
0	0	0	0	0	0	1	0	1
0	1	1	1	1	0	0	0	1
0	0	1	1	0	0	1	0	1
0	1	1	1	1	1	1	1	1
0	1	1	1	1	0	0	1	1
0	1	1	1	0	0	0	0	1
0	1	1	1	1	1	1	0	1
0	0	0	0	0	1	0	1	1
0	0	0	0	0	1	0	1	1
0	1	0	0	1	1	0	1	1
0	1	0	0	1	1	0	1	1
0	0	0	0	0	0	0	0	0
0	0	0	0	0	0	0	0	0
0	0	0	0	0	0	0	0	1
0	1	0	0	1	1	0	1	1
0	0	0	0	0	0	0	0	0
0	0	0	0	0	0	0	0	0
0	1	1	1	1	1	0	1	1
0	1	0	1	1	1	0	0	1
0	1	1	1	1	1	1	1	1
0	0	0	1	0	1	0	1	1
0	0	0	1	0	0	0	0	0
0	0	0	0	0	0	0	0	0
0	0	0	0	0	0	0	0	1
0	1	0	1	1	1	0	1	1
0	1	0	0	1	1	0	1	1
0	0	0	0	0	0	0	0	1
0	0	0	0	0	0	0	0	1

0	0	0	0	0	0	0	0	1
0	0	0	0	0	0	0	1	1
0	0	0	0	0	0	0	0	1
0	0	0	0	0	0	0	0	1
0	0	0	0	0	0	0	0	1
0	0	0	0	0	0	0	1	1
0	1	0	0	1	1	0	1	1
0	0	0	0	0	1	0	1	1
0	1	0	0	1	1	0	1	1
0	0	0	1	0	0	0	0	1
0	1	0	0	1	1	0	1	1
0	0	0	0	1	1	0	1	1
0	0	0	0	0	0	0	0	1
0	1	1	0	1	1	1	1	1
0	1	1	0	1	1	0	1	1
0	1	0	0	1	1	0	1	1
0	1	0	0	1	1	0	1	1
0	1	0	0	0	1	0	0	1
0	1	0	0	1	1	0	1	1
0	0	0	0	0	0	0	0	1
0	0	0	0	0	0	0	1	1
0	0	0	0	0	0	0	1	1
0	0	0	0	0	0	0	0	1
0	1	0	0	1	1	0	1	1
0	0	0	0	0	0	0	0	1
0	0	0	0	0	0	0	0	1
0	0	0	0	0	0	0	0	1
0	0	0	0	0	0	0	0	1
0	0	0	0	0	0	0	0	1
0	0	0	0	0	0	0	0	1
0	0	0	0	0	0	0	0	1
0	0	0	0	0	1	1	1	1
0	0	0	0	0	0	0	0	0
0	1	0	0	1	1	0	1	1
0	1	0	0	1	1	0	1	1
0	1	0	0	1	1	0	1	1
0	0	0	0	0	0	0	0	1
0	0	0	0	1	1	0	1	1
0	1	1	1	1	1	0	1	1
0	1	0	0	1	1	0	1	1
0	1	1	0	1	1	0	1	1
0	0	0	0	0	0	0	1	1
0	0	0	0	0	0	1	1	1
0	0	0	0	1	1	0	1	1
0	1	0	0	1	1	0	1	1
0	0	0	0	0	0	0	0	1
0	0	0	0	0	0	0	0	0
0	0	0	0	0	0	0	0	1
0	0	0	0	0	0	0	0	1
0	1	0	0	1	1	0	1	1

0	1	1	0	1	1	0	1	1
0	1	0	0	1	1	0	1	1
0	1	1	0	1	1	0	1	1

OHL_13	OHL_14	OHL_15	OHL_total	OHL_read	OHL_count	OHL_listen	OHL_deal	tquality_1
1	0	0	7	0.33	0.75	0.00	0.40	0
1	1	1	9	0.17	0.25	1.00	1.00	0
1	0	1	11	0.50	0.50	1.00	0.80	0
1	0	1	14	1.00	1.00	0.00	0.80	0
1	0	0	10	0.67	0.25	1.00	0.60	0
1	0	0	10	0.67	0.25	1.00	0.60	0
1	0	1	11	0.50	0.50	1.00	0.80	0
1	0	1	12	0.67	0.75	0.50	0.80	0
1	0	1	12	0.67	0.75	0.50	0.80	0
1	0	1	14	0.83	1.00	0.50	0.80	0
1	1	1	14	0.83	0.75	0.50	1.00	0
1	0	1	14	0.83	1.00	0.50	0.80	0
1	1	0	13	0.83	1.00	0.00	0.80	0
1	1	0	13	0.50	1.00	1.00	0.80	1
1	0	0	6	0.17	0.50	0.50	0.40	0
1	0	0	12	0.67	0.75	1.00	0.60	0
1	0	1	12	0.83	0.75	0.00	0.80	0
1	0	1	12	0.83	1.00	0.00	0.60	0
1	0	1	9	0.50	0.50	0.00	0.80	0
1	0	0	6	0.33	0.00	0.50	0.60	0
1	1	1	15	0.67	1.00	1.00	1.00	0
1	1	1	15	0.67	1.00	1.00	1.00	0
1	1	1	16	0.83	1.00	1.00	1.00	0
1	1	1	16	0.83	1.00	1.00	1.00	0
0	0	1	9	0.67	0.25	0.50	0.60	0
1	0	1	10	0.50	0.50	0.50	0.80	0
0	0	1	6	0.50	0.25	0.00	0.40	0
1	1	1	15	0.67	1.00	1.00	1.00	0
1	1	0	11	0.33	0.75	1.00	0.80	0
1	0	1	13	0.83	0.75	1.00	0.60	0
1	1	1	14	0.50	1.00	1.00	1.00	0
1	1	0	12	0.50	1.00	1.00	0.60	0
1	0	1	11	0.17	1.00	1.00	0.80	0
0	0	1	7	0.33	0.25	1.00	0.40	0
1	1	0	11	0.50	0.75	1.00	0.60	0
1	0	0	10	0.50	0.75	1.00	0.40	0
1	0	0	10	0.67	0.50	1.00	0.40	0
1	0	0	8	0.50	0.25	1.00	0.40	0
1	0	0	8	0.50	0.25	1.00	0.40	0
1	1	0	16	1.00	1.00	1.00	0.80	0
1	0	0	8	0.33	0.50	0.50	0.60	0
1	0	1	11	0.50	0.75	1.00	0.60	0
1	0	1	11	0.33	0.75	1.00	0.80	0
1	1	0	13	0.67	1.00	0.50	0.80	0
1	0	1	16	1.00	1.00	1.00	0.80	0
1	0	0	10	0.33	1.00	0.50	0.60	0
1	0	1	8	0.50	0.25	0.00	0.80	0
1	0	1	11	0.50	0.50	1.00	0.80	0
1	1	0	7	0.17	0.75	0.00	0.60	0

1	1	0	12	0.50	0.75	1.00	0.80	1
1	1	0	13	0.67	0.75	1.00	0.80	0
1	0	0	11	0.83	0.50	1.00	0.40	0
1	1	0	14	0.83	0.75	1.00	0.80	0
1	0	1	15	0.83	1.00	1.00	0.80	0
1	1	0	16	1.00	1.00	1.00	0.80	0
0	0	0	7	0.33	0.50	0.50	0.40	0
1	0	1	11	0.67	0.50	1.00	0.60	0
1	0	1	12	0.50	0.75	1.00	0.80	0
1	0	1	12	0.50	0.75	1.00	0.80	0
1	0	0	9	0.50	0.50	1.00	0.40	0
1	1	1	13	0.33	1.00	1.00	1.00	0
1	1	1	16	1.00	1.00	1.00	0.80	0
1	0	1	5	0.00	0.25	0.50	0.60	0
1	1	1	12	0.50	0.50	1.00	1.00	0
1	1	1	15	0.83	1.00	0.50	1.00	0
1	1	1	16	0.83	1.00	1.00	1.00	0
1	0	0	9	0.67	0.50	0.50	0.40	0
1	1	0	13	0.50	1.00	1.00	0.80	0
1	1	1	16	0.83	1.00	1.00	1.00	0
1	0	1	14	0.67	1.00	1.00	0.80	0
1	0	1	14	0.67	1.00	1.00	0.80	0
0	0	0	6	0.17	0.25	1.00	0.40	0
1	0	0	8	0.33	0.50	0.50	0.60	0
1	1	1	14	0.50	1.00	1.00	1.00	0
1	1	0	7	0.17	0.75	0.00	0.60	0
1	1	0	10	0.33	0.75	1.00	0.60	0
0	0	0	3	0.17	0.00	0.00	0.40	0
1	0	1	11	0.67	0.50	1.00	0.60	0
1	0	1	16	1.00	1.00	1.00	0.80	0
1	1	1	15	0.83	0.75	1.00	1.00	0
1	0	0	13	0.83	0.75	1.00	0.60	0
1	0	0	9	0.33	0.50	1.00	0.60	0
1	0	0	11	0.50	0.75	1.00	0.60	0
0	0	0	3	0.00	0.25	0.50	0.20	0
1	1	1	12	0.33	1.00	0.50	1.00	0
0	0	0	5	0.17	0.25	0.50	0.40	0
0	0	0	7	0.33	0.50	0.50	0.40	0
1	0	0	9	0.67	0.25	0.50	0.60	0
1	1	0	8	0.33	0.00	1.00	0.80	0
1	0	0	7	0.17	0.50	1.00	0.40	0
1	0	1	14	0.67	1.00	1.00	0.80	0
1	0	0	8	0.50	0.25	1.00	0.40	0
1	0	1	15	0.83	1.00	1.00	0.80	0
1	1	1	15	0.83	1.00	1.00	0.80	0
1	0	0	7	0.33	0.50	0.50	0.40	0
1	1	1	14	0.67	0.75	1.00	1.00	0
0	0	0	5	0.17	0.25	1.00	0.20	0
1	0	1	11	0.67	0.50	0.50	0.80	0
0	0	0	6	0.50	0.25	0.50	0.20	0

1	1	0	10	0.67	0.50	0.50	0.60	0
0	1	1	13	0.50	1.00	1.00	0.80	0
0	1	1	13	0.50	1.00	1.00	0.80	0
1	0	1	9	0.33	0.50	1.00	0.60	0
1	1	0	13	0.50	1.00	1.00	0.80	0
1	1	1	13	0.67	0.75	0.50	1.00	0
0	0	0	8	0.17	1.00	1.00	0.20	0
1	1	1	10	0.17	1.00	0.50	0.80	0
1	1	1	13	0.67	1.00	0.00	1.00	0
1	1	0	9	0.50	0.25	0.50	0.80	0
1	1	0	14	0.67	1.00	1.00	0.80	0
1	1	0	14	0.67	1.00	1.00	0.80	0
0	1	0	9	0.67	0.25	1.00	0.40	0
1	1	1	14	0.67	0.75	1.00	1.00	0
1	1	1	15	0.67	1.00	1.00	1.00	0
1	1	1	6	0.00	0.25	0.00	1.00	0
1	0	0	9	0.50	0.75	0.00	0.60	0
0	0	1	8	0.33	0.75	0.50	0.40	0
0	1	0	8	0.50	0.50	0.50	0.40	0
1	0	1	16	1.00	1.00	1.00	0.80	0
1	0	0	9	0.50	0.75	0.50	0.40	0
0	1	0	4	0.17	0.25	0.00	0.40	0
1	0	0	7	0.67	0.00	0.50	0.40	0
1	0	0	8	0.33	1.00	0.00	0.40	0
0	0	0	5	0.17	0.50	0.50	0.20	0
1	0	1	12	0.33	1.00	1.00	0.80	0
1	1	0	10	0.33	1.00	0.00	0.80	0
1	1	0	9	0.50	0.75	0.00	0.60	0
1	1	0	12	0.50	1.00	1.00	0.60	0
1	0	0	7	0.50	0.00	0.50	0.60	0
1	0	1	8	0.50	0.00	0.50	0.80	0
1	0	1	10	0.50	0.50	0.50	0.80	0
1	0	1	11	0.67	0.50	0.50	0.80	0
0	0	0	1	0.17	0.00	0.00	0.00	0
0	0	0	1	0.17	0.00	0.00	0.00	0
1	0	0	3	0.17	0.00	0.00	0.40	0
1	0	1	11	0.67	0.50	0.50	0.80	0
0	0	0	3	0.50	0.00	0.00	0.00	0
0	0	0	4	0.67	0.00	0.00	0.00	0
1	1	1	13	0.50	1.00	0.50	1.00	0
1	0	1	11	0.67	0.75	0.50	0.60	0
0	0	1	14	0.83	1.00	1.00	0.60	0
1	0	0	7	0.33	0.25	0.50	0.60	0
1	0	0	3	0.17	0.25	0.00	0.20	0
0	0	0	1	0.17	0.00	0.00	0.00	0
1	0	0	3	0.17	0.00	0.00	0.40	0
1	1	1	13	0.67	0.75	0.50	1.00	0
1	0	0	8	0.33	0.50	0.50	0.60	0
0	0	0	1	0.00	0.00	0.00	0.20	0
0	0	0	3	0.33	0.00	0.00	0.20	0

0	0	0	2	0.17	0.00	0.00	0.20	0
0	0	0	4	0.33	0.00	0.00	0.40	0
0	0	0	3	0.33	0.00	0.00	0.20	0
0	0	0	2	0.17	0.00	0.00	0.20	0
0	0	0	2	0.17	0.00	0.00	0.20	0
1	0	1	7	0.50	0.00	0.00	0.80	0
0	0	1	10	0.67	0.50	0.50	0.60	0
1	0	0	7	0.50	0.00	0.50	0.60	0
1	0	1	11	0.67	0.50	0.50	0.80	0
1	0	0	5	0.33	0.25	0.00	0.40	0
1	0	1	11	0.67	0.50	0.50	0.80	0
1	0	1	8	0.33	0.25	0.50	0.80	0
1	0	0	4	0.33	0.00	0.00	0.40	0
1	0	1	13	0.67	0.75	1.00	0.80	0
1	0	1	12	0.67	0.75	0.50	0.80	0
1	0	1	11	0.67	0.50	0.50	0.80	0
1	0	1	10	0.50	0.50	0.50	0.80	0
1	0	0	8	0.67	0.25	0.50	0.40	0
1	0	1	10	0.50	0.50	0.50	0.80	0
1	0	0	4	0.33	0.00	0.00	0.40	0
1	0	0	6	0.50	0.00	0.00	0.60	0
1	0	0	5	0.33	0.00	0.00	0.60	0
1	0	0	4	0.33	0.00	0.00	0.40	0
1	0	1	10	0.50	0.50	0.50	0.80	0
1	0	0	5	0.50	0.00	0.00	0.40	0
0	0	0	3	0.33	0.00	0.00	0.20	0
0	0	0	3	0.33	0.00	0.00	0.20	0
0	0	0	3	0.33	0.00	0.00	0.20	0
0	0	0	4	0.50	0.00	0.00	0.20	0
0	0	0	3	0.33	0.00	0.00	0.20	0
0	0	0	2	0.17	0.00	0.00	0.20	0
1	0	0	9	0.67	0.00	1.00	0.60	0
0	0	0	0	0.00	0.00	0.00	0.00	0
1	0	0	10	0.67	0.50	0.50	0.60	0
1	0	1	10	0.50	0.50	0.50	0.80	0
1	0	1	11	0.67	0.50	0.50	0.80	0
0	0	0	3	0.33	0.00	0.00	0.20	0
1	0	1	10	0.67	0.25	0.50	0.80	0
1	1	1	14	0.67	1.00	0.50	1.00	0
1	0	1	11	0.67	0.50	0.50	0.80	0
1	0	1	12	0.67	0.75	0.50	0.80	0
0	0	0	4	0.33	0.00	0.00	0.40	0
1	0	1	8	0.50	0.00	0.50	0.80	0
1	0	1	9	0.50	0.25	0.50	0.80	0
1	0	1	11	0.67	0.50	0.50	0.80	0
0	0	0	2	0.17	0.00	0.00	0.20	0
0	0	0	1	0.17	0.00	0.00	0.00	0
0	0	0	3	0.33	0.00	0.00	0.20	0
0	0	0	3	0.33	0.00	0.00	0.20	0
1	0	1	10	0.50	0.50	0.50	0.80	0

1	0	1	12	0.67	0.75	0.50	0.80	0
1	0	1	11	0.67	0.50	0.50	0.80	0
1	0	1	12	0.67	0.75	0.50	0.80	0

tquality_2	tquality_3	tquality_4	tquality_5	tquality_6	tquality_7	tquality_8	tquality_9	tquality_10
0	0	0	0	0	0	0	0	0
0	0	0	0	0	0	0	0	0
0	0	0	0	0	0	0	0	0
0	0	0	0	0	0	0	0	0
0	0	0	0	0	0	0	0	0
0	0	0	0	0	0	0	0	0
0	0	0	0	0	0	0	0	0
0	0	0	0	0	0	0	0	0
0	0	0	0	0	0	0	0	0
0	0	0	0	0	0	0	0	0
0	0	0	0	0	0	0	0	0
0	0	0	0	0	0	0	0	0
0	0	0	0	0	0	0	0	0
0	0	0	0	0	0	0	0	0
0	0	0	0	0	0	0	0	0
0	0	0	0	0	0	0	0	0
0	0	0	0	0	0	0	0	0
0	0	0	0	0	0	0	0	0
0	0	0	0	0	0	0	0	0
0	0	0	0	0	0	0	0	0
0	0	0	0	0	0	0	0	0
0	0	0	1	0	0	0	0	0
0	0	0	0	0	0	0	0	0
0	0	0	0	0	0	0	0	0
0	0	0	0	0	0	0	0	0
0	0	0	0	0	0	0	0	0
0	0	0	0	0	0	0	0	0
0	0	0	0	0	0	0	0	0
0	0	0	0	1	0	0	0	0
0	0	0	0	0	0	0	0	0
0	0	0	0	0	0	0	0	0
0	0	1	1	1	1	0	0	1
0	0	0	0	0	0	0	0	0
0	0	0	0	0	0	0	0	0
0	0	0	0	0	0	0	0	0
0	0	1	0	0	0	0	0	1
0	0	0	0	0	0	0	0	0
0	0	0	0	0	0	0	0	0
0	0	0	0	0	0	0	0	0
0	0	0	0	0	0	0	0	0
0	0	0	0	0	0	0	0	0
0	0	0	0	0	0	0	0	0
0	0	0	0	0	0	0	0	0
0	0	0	0	0	1	0	0	1
0	0	0	0	0	0	0	0	0
0	0	0	0	0	0	0	0	0
0	0	0	0	0	0	0	0	0
0	0	0	0	0	0	0	0	0
0	0	0	0	0	0	0	0	0

1	0	0	0	0	0	0	1	0
0	0	0	0	0	0	0	0	0
0	0	0	0	0	0	0	0	0
0	0	0	0	0	0	0	0	0
0	0	0	0	0	0	0	0	0
0	0	0	0	0	0	0	0	0
0	0	0	0	0	0	0	0	0
0	0	0	0	0	0	0	0	0
0	0	0	0	0	0	0	0	0
0	0	0	0	0	0	0	0	0
0	0	0	0	0	0	0	0	0
0	0	0	0	0	0	0	0	0
0	0	0	0	0	0	0	0	0
0	0	0	0	0	0	0	0	0
0	0	0	0	0	0	0	0	0
0	0	0	0	0	0	0	0	0
0	0	0	0	0	0	0	0	0
0	0	0	0	1	0	0	0	0
0	1	0	0	0	0	0	0	0
0	0	0	0	0	0	0	0	0
0	0	0	0	0	0	0	0	0
0	0	0	0	0	0	0	0	0
0	0	0	0	0	0	0	0	0
0	0	0	0	0	0	0	0	0
0	0	0	0	0	0	0	0	0
0	0	0	0	0	0	0	0	0
0	0	0	0	0	0	0	0	0
0	0	0	0	0	0	0	0	0
0	0	0	0	0	0	0	0	0
0	0	0	0	0	0	0	0	0
0	0	0	0	0	0	0	0	0
0	0	0	0	0	0	0	0	0
0	0	0	0	0	0	0	0	0
0	0	0	0	0	0	0	0	0
0	0	0	0	0	0	0	0	0
0	0	0	0	0	0	0	0	0
0	0	0	0	0	0	0	0	0
0	0	0	0	0	0	0	0	0
0	0	0	0	0	0	0	0	0
0	0	0	0	0	0	0	0	0
0	0	0	0	0	0	0	0	0
0	0	0	0	0	0	0	0	0
0	0	0	0	0	0	0	0	0
0	0	0	0	0	0	0	1	0
1	0	1	1	0	1	0	0	0
0	0	0	1	1	0	0	0	1
0	0	0	0	0	0	0	0	0
0	0	0	0	0	0	0	0	0
0	0	0	0	0	0	0	0	0
0	0	0	0	0	0	0	0	0

0	0	0	0	0	0	0	0	0
0	0	0	0	0	0	0	0	0
0	0	0	0	0	0	0	0	0
0	0	0	0	0	0	0	0	0
0	0	0	1	1	0	0	0	0
0	0	0	0	0	0	0	0	0
0	0	0	0	0	0	0	0	0
0	0	0	0	0	0	0	0	0
0	0	0	0	0	0	0	0	0
0	0	0	0	0	0	0	0	0
0	0	0	0	0	0	0	0	0
0	0	0	0	0	0	0	0	0
0	0	0	0	0	0	0	0	0
0	0	0	0	0	0	0	0	0
0	0	0	0	0	0	0	0	0
0	0	0	0	0	0	0	0	0
0	0	0	0	0	0	0	0	0
0	0	0	0	0	0	0	0	0
0	0	0	0	0	0	0	0	0
0	0	0	1	0	0	0	0	0
0	0	0	0	0	0	0	0	0
0	0	0	0	0	0	0	0	0
0	0	0	0	0	0	0	0	0
0	0	0	0	1	0	0	0	0
0	0	0	0	0	0	0	0	0
0	0	0	0	0	0	0	0	0
0	0	0	0	0	0	0	0	0
0	0	0	0	0	0	0	0	0
0	0	0	0	0	0	0	0	0
0	0	0	0	0	0	0	0	0
0	0	0	0	0	0	0	0	0
0	0	0	0	0	0	0	0	0
0	0	0	0	0	0	0	0	0
0	0	0	0	0	0	0	0	0
0	0	0	0	0	0	0	0	0
0	0	0	0	0	0	0	0	0
0	0	0	0	0	0	0	0	0
0	0	0	0	0	0	0	0	0
0	0	0	0	0	0	0	0	0
0	0	0	0	0	0	0	0	0
0	0	0	0	0	0	0	0	0
0	0	0	0	0	0	0	0	0
0	0	0	0	0	0	0	0	0
0	0	0	0	0	0	0	0	0
0	0	0	0	0	0	0	0	0
0	0	0	0	0	0	0	0	0
0	0	0	0	0	0	0	0	0
0	0	0	0	0	0	0	0	0
0	0	0	0	0	0	0	0	0
0	0	0	0	0	0	0	0	0

0	0	0	0	0	0	0	0	0
0	0	0	0	0	0	0	0	0
0	0	0	0	0	0	0	0	0
0	0	0	0	0	0	0	0	0
0	0	0	0	0	0	0	0	0
0	0	0	0	0	0	0	0	0
0	0	0	0	0	0	0	0	0
0	0	0	0	0	0	0	0	0
0	0	0	0	0	0	0	0	0
0	0	0	0	0	0	0	0	0
0	0	0	0	0	0	0	0	0
0	0	0	0	0	0	0	0	0
0	0	0	0	0	0	0	0	0
0	0	0	0	0	0	0	0	0
0	0	0	0	0	0	0	0	0
0	0	0	0	0	0	0	0	0
0	0	0	0	0	0	0	0	0
0	0	0	0	0	0	0	0	0
0	0	0	0	0	0	0	0	0
0	0	0	0	0	0	0	0	0
0	0	0	0	0	0	0	0	0
0	0	0	0	0	0	0	0	0
0	0	0	0	0	0	0	0	0
0	0	0	0	0	0	0	0	0
0	0	0	0	0	0	0	0	0
0	0	0	0	0	0	0	0	0
0	0	0	0	0	0	0	0	0
0	0	0	0	0	0	0	0	0
0	0	0	0	0	0	0	0	0
0	0	0	0	0	0	0	0	0
0	0	0	0	0	0	0	0	0
0	0	0	0	0	0	0	0	0
0	0	0	0	0	0	0	0	0
0	0	0	0	0	0	0	0	0
0	0	0	0	0	0	0	0	0
0	0	0	0	0	0	0	0	0
0	0	0	0	0	0	0	0	0
0	0	0	0	0	0	0	0	0
0	0	0	0	0	0	0	0	0
0	0	0	0	0	0	0	0	0
0	0	0	0	0	0	0	0	0
0	0	0	0	0	0	0	0	0
0	0	0	0	0	0	0	0	0
0	0	0	0	0	0	0	0	0
0	0	0	0	0	0	0	0	0
0	0	0	0	0	0	0	0	0
0	0	0	0	0	0	0	0	0
0	0	0	0	0	0	0	0	0
0	0	0	0	0	0	0	0	0
0	0	0	0	0	0	0	0	0

0	0	0	0	0	0	0	0	0
0	0	0	0	0	0	0	0	0
0	0	0	0	0	0	0	0	0

tquality_11	tquality_12	tquality_13	tquality_14	tquality_to	quality_po	quality_scc	quality平方	quality_scc
0	0	0	0	0	1	1.00	5.00	1.00
0	0	0	0	0	1	1.00	4.58	1.00
0	0	0	0	0	1	1.00	1.73	0.00
0	0	0	0	0	1	1.00	2.83	1.00
0	0	0	0	0	1	0.00	1.00	0.00
0	0	0	0	0	0	0.00	0.00	0.00
0	0	0	0	0	1	1.00	1.73	0.00
0	0	0	0	0	0	0.00	0.00	0.00
0	0	0	0	0	1	1.00	2.65	1.00
0	0	0	0	0	0	0.00	0.00	0.00
0	0	0	0	0	1	1.00	3.16	1.00
0	0	0	0	0	1	1.00	1.41	0.00
0	0	0	0	0	0	0.00	0.00	0.00
0	0	0	0	1	1	1.00	3.61	1.00
0	0	0	0	0	0	0.00	0.00	0.00
0	0	0	0	0	1	1.00	1.41	0.00
0	0	0	0	0	0	0.00	0.00	0.00
0	0	0	0	0	0	0.00	0.00	0.00
0	0	0	0	0	1	1.00	3.61	1.00
0	0	0	0	0	1	1.00	3.74	1.00
0	0	0	0	0	1	1.00	1.41	0.00
0	0	0	0	1	1	1.00	3.16	1.00
0	0	0	0	0	0	0.00	0.00	0.00
0	0	0	0	0	1	1.00	2.24	1.00
0	0	0	0	0	1	1.00	1.41	0.00
0	0	0	0	0	0	0.00	0.00	0.00
0	0	0	0	0	1	1.00	3.87	1.00
0	0	0	0	0	1	1.00	4.00	1.00
0	0	0	0	1	1	1.00	4.36	1.00
0	0	0	0	0	1	1.00	3.32	1.00
0	0	0	0	0	1	1.00	3.74	1.00
0	0	0	0	5	1	1.00	5.57	1.00
0	0	0	0	0	1	1.00	2.00	1.00
0	0	0	0	0	1	1.00	3.16	1.00
0	0	0	0	0	1	1.00	2.83	1.00
0	0	0	0	2	1	1.00	5.29	1.00
0	0	0	0	0	1	1.00	2.65	1.00
0	0	0	0	0	1	1.00	3.87	1.00
0	0	1	0	1	1	1.00	4.90	1.00
0	0	0	0	0	0	0.00	0.00	0.00
0	0	0	0	0	1	1.00	3.87	1.00
0	0	0	0	0	0	0.00	0.00	0.00
0	0	0	0	0	1	1.00	3.16	1.00
0	0	0	0	2	1	1.00	3.61	1.00
0	0	0	0	0	1	1.00	1.41	0.00
0	0	0	0	0	1	1.00	2.45	1.00
0	0	0	0	0	1	1.00	4.12	1.00
0	0	0	0	0	1	1.00	4.58	1.00
0	0	0	0	0	1	1.00	4.00	1.00

0	0	0	0	3	1	1.00	5.20	1.00
0	0	0	0	0	1	1.00	1.41	0.00
0	0	0	0	0	1	1.00	4.36	1.00
0	0	0	0	0	0	0.00	0.00	0.00
0	0	0	0	0	1	1.00	1.41	0.00
0	0	0	0	0	1	1.00	4.24	1.00
0	0	0	0	0	1	1.00	2.45	1.00
0	0	0	0	0	0	0.00	0.00	0.00
0	0	0	0	0	1	1.00	4.47	1.00
0	0	0	0	0	1	1.00	4.47	1.00
0	0	0	0	0	1	1.00	3.87	1.00
0	0	0	0	0	1	1.00	2.45	1.00
0	0	0	0	0	1	1.00	3.00	1.00
0	0	0	0	0	1	1.00	4.24	1.00
0	0	0	0	0	1	1.00	3.74	1.00
0	0	0	0	0	1	1.00	4.12	1.00
0	0	0	0	0	1	1.00	2.24	1.00
0	0	0	0	1	1	1.00	2.83	1.00
0	0	0	0	1	1	1.00	4.12	1.00
0	0	0	0	0	1	1.00	4.69	1.00
0	0	0	0	0	1	1.00	3.74	1.00
0	0	0	0	0	0	0.00	0.00	0.00
0	0	0	0	0	1	1.00	2.45	1.00
0	0	0	0	0	1	1.00	2.00	1.00
0	0	0	0	0	1	1.00	1.41	0.00
0	0	0	0	0	1	0.00	1.00	0.00
0	0	0	0	0	1	1.00	1.41	0.00
0	0	0	0	0	0	0.00	0.00	0.00
0	0	0	0	0	0	0.00	0.00	0.00
0	0	0	0	0	0	0.00	0.00	0.00
0	0	0	0	0	1	1.00	2.83	1.00
0	0	0	0	0	1	0.00	1.00	0.00
0	0	0	0	0	1	1.00	3.32	1.00
0	0	0	0	0	1	0.00	1.00	0.00
0	0	0	0	0	1	1.00	1.41	0.00
0	0	0	0	0	0	0.00	0.00	0.00
0	0	0	0	0	1	1.00	4.58	1.00
0	0	0	0	0	1	0.00	1.00	0.00
0	0	0	0	0	1	1.00	1.73	0.00
0	0	0	0	0	0	0.00	0.00	0.00
0	0	0	0	0	1	1.00	2.00	1.00
0	0	0	0	0	0	0.00	0.00	0.00
0	0	0	0	0	1	1.00	3.16	1.00
0	0	0	0	1	1	1.00	4.80	1.00
0	0	0	0	4	1	1.00	5.10	1.00
0	0	1	0	4	1	1.00	4.24	1.00
0	0	0	0	0	1	1.00	1.73	0.00
0	0	0	0	0	1	1.00	2.24	1.00
0	0	0	0	0	0	0.00	0.00	0.00
0	0	0	0	0	1	1.00	2.65	1.00

0	0	0	0	0	0	0.00	0.00	0.00
0	0	0	0	0	1	1.00	3.87	1.00
0	0	0	0	0	1	1.00	3.32	1.00
0	0	0	0	0	1	1.00	2.00	1.00
0	0	0	0	2	1	1.00	3.61	1.00
0	0	0	0	0	1	1.00	3.32	1.00
0	0	0	0	0	1	1.00	1.41	0.00
0	0	0	0	0	1	1.00	1.73	0.00
0	0	0	0	0	1	1.00	2.00	1.00
0	0	0	0	0	0	0.00	0.00	0.00
0	0	0	0	0	0	0.00	0.00	0.00
0	0	0	0	0	0	0.00	0.00	0.00
0	0	0	0	0	1	1.00	3.87	1.00
0	0	0	0	0	1	1.00	4.00	1.00
0	0	0	0	0	1	1.00	3.74	1.00
0	0	0	0	0	1	1.00	1.73	0.00
0	0	0	0	0	1	0.00	1.00	0.00
0	0	0	0	0	1	1.00	1.73	0.00
0	0	0	0	0	1	1.00	1.73	0.00
0	0	0	0	1	1	1.00	2.65	1.00
0	0	0	0	0	1	1.00	1.73	0.00
0	0	0	0	0	1	1.00	1.41	0.00
0	0	0	0	0	1	1.00	2.24	1.00
0	0	0	0	1	1	1.00	2.45	1.00
0	0	0	0	0	1	0.00	1.00	0.00
0	0	0	0	0	1	1.00	2.00	1.00
0	0	0	0	0	1	1.00	1.73	0.00
0	0	0	0	0	1	1.00	1.41	0.00
0	0	0	0	0	1	1.00	1.41	0.00
0	0	0	0	0	1	1.00	2.00	1.00
0	0	0	0	0	0	0.00	0.00	0.00
0	0	0	0	0	1	1.00	3.74	1.00
0	0	0	0	0	0	0.00	0.00	0.00
0	0	0	0	0	1	1.00	3.74	1.00
0	0	0	0	0	0	0.00	0.00	0.00
0	0	0	0	0	0	0.00	0.00	0.00
0	0	0	0	0	0	0.00	0.00	0.00
0	0	0	0	0	0	0.00	0.00	0.00
0	0	0	0	0	1	1.00	3.74	1.00
0	0	0	0	0	1	1.00	2.45	1.00
0	0	0	0	0	1	1.00	3.32	1.00
0	0	0	0	0	0	0.00	0.00	0.00
0	0	0	0	0	1	1.00	4.00	1.00
0	0	0	0	0	1	1.00	5.20	1.00
0	0	0	0	0	1	1.00	3.74	1.00
0	0	0	0	0	1	1.00	4.58	1.00
0	0	0	0	0	1	1.00	3.74	1.00
0	0	0	0	0	0	0.00	0.00	0.00
0	0	0	0	0	1	1.00	3.74	1.00
0	0	0	0	0	1	1.00	3.74	1.00

0	0	0	0	0	0	0.00	0.00	0.00
0	0	0	0	0	0	0.00	0.00	0.00
0	0	0	0	0	1	1.00	1.73	0.00
0	0	0	0	0	0	0.00	0.00	0.00
0	0	0	0	0	0	0.00	0.00	0.00
0	0	0	0	0	1	1.00	3.74	1.00
0	0	0	0	0	1	1.00	4.58	1.00
0	0	0	0	0	1	0.00	1.00	0.00
0	0	0	0	0	1	1.00	3.61	1.00
0	0	0	0	0	1	1.00	1.41	0.00
0	0	0	0	0	0	0.00	0.00	0.00
0	0	0	0	0	0	0.00	0.00	0.00
0	0	0	0	0	0	0.00	0.00	0.00
0	0	0	0	0	0	0.00	0.00	0.00
0	0	0	0	0	0	0.00	0.00	0.00
0	0	0	0	0	0	0.00	0.00	0.00
0	0	0	0	0	0	0.00	0.00	0.00
0	0	0	0	0	0	0.00	0.00	0.00
0	0	0	0	0	0	0.00	0.00	0.00
0	0	0	0	0	0	0.00	0.00	0.00
0	0	0	0	0	1	1.00	3.74	1.00
0	0	0	0	0	1	1.00	3.74	1.00
0	0	0	0	0	1	1.00	3.87	1.00
0	0	0	0	0	1	1.00	3.00	1.00
0	0	0	0	0	0	0.00	0.00	0.00
0	0	0	0	0	1	1.00	3.74	1.00
0	0	0	0	0	1	1.00	3.74	1.00
0	0	0	0	0	1	1.00	3.74	1.00
0	0	0	0	0	0	0.00	0.00	0.00
0	0	0	0	0	1	1.00	3.74	1.00
0	0	0	0	0	0	0.00	0.00	0.00
0	0	0	0	0	0	0.00	0.00	0.00
0	0	0	0	0	0	0.00	0.00	0.00
0	0	0	0	0	1	1.00	3.32	1.00
0	0	0	0	0	0	0.00	0.00	0.00
0	0	0	0	0	0	0.00	0.00	0.00
0	0	0	0	0	0	0.00	0.00	0.00
0	0	0	0	0	0	0.00	0.00	0.00
0	0	0	0	0	1	1.00	3.46	1.00
0	0	0	0	0	1	1.00	3.16	1.00
0	0	0	0	0	1	1.00	3.74	1.00
0	0	0	0	0	0	0.00	0.00	0.00
0	0	0	0	0	0	0.00	0.00	0.00
0	0	0	0	0	0	0.00	0.00	0.00
0	0	0	0	0	0	0.00	0.00	0.00
0	0	0	0	0	0	0.00	0.00	0.00
0	0	0	0	0	0	0.00	0.00	0.00
0	0	0	0	0	0	0.00	0.00	0.00
0	0	0	0	0	0	0.00	0.00	0.00
0	0	0	0	0	0	0.00	0.00	0.00

0	0	0	0	0	0	0.00	0.00	0.00
0	0	0	0	0	0	0.00	0.00	0.00
0	0	0	0	0	0	0.00	0.00	0.00

quality_scc	edu1	edu2	edu3	edu4	edu5	income1	income2	edu_r
1.00	1	0	0	0	0	0	0	1
1.00	0	0	0	0	0	0	0	1
0.00	0	0	1	0	0	0	0	2
1.00	0	0	1	0	0	1	0	2
0.00	0	1	0	0	0	1	0	1
0.00	0	1	0	0	0	0	0	1
0.00	1	0	0	0	0	0	0	1
0.00	0	0	1	0	0	1	0	2
1.00	0	0	1	0	0	1	0	2
0.00	0	0	0	0	1	0	1	2
1.00	1	0	0	0	0	1	0	1
0.00	0	0	0	1	0	1	0	2
0.00	0	1	0	0	0	1	0	1
1.00	1	0	0	0	0	0	0	1
0.00	0	0	0	1	0	0	0	2
0.00	1	0	0	0	0	0	0	1
0.00	1	0	0	0	0	0	0	1
0.00	0	0	0	1	0	0	0	2
1.00	1	0	0	0	0	0	0	1
1.00	0	0	1	0	0	0	1	2
0.00	0	1	0	0	0	1	0	1
1.00	0	0	0	1	0	0	1	2
0.00	0	0	1	0	0	0	1	2
0.00	0	0	1	0	0	0	1	2
0.00	1	0	0	0	0	0	0	1
0.00	1	0	0	0	0	0	0	1
1.00	0	0	1	0	0	0	0	2
1.00	0	0	0	1	0	0	1	2
1.00	0	0	0	0	1	0	1	2
1.00	0	0	0	1	0	1	0	2
1.00	0	0	1	0	0	1	0	2
1.00	0	0	0	0	1	0	1	2
0.00	0	0	0	1	0	0	1	2
1.00	0	0	1	0	0	0	1	2
1.00	0	0	0	0	0	1	0	1
1.00	0	0	1	0	0	0	1	2
1.00	0	0	1	0	0	1	0	2
1.00	0	0	1	0	0	1	0	2
1.00	1	0	0	0	0	1	0	1
0.00	0	0	0	0	1	0	1	2
1.00	0	1	0	0	0	0	0	1
0.00	0	0	1	0	0	1	0	2
1.00	0	0	1	0	0	0	1	2
1.00	0	0	0	1	0	0	0	2
0.00	0	0	0	0	1	0	1	2
1.00	1	0	0	0	0	1	0	1
1.00	0	0	0	0	1	0	1	2
1.00	0	1	0	0	0	0	0	1
1.00	0	1	0	0	0	1	0	1

1.00	0	0	0	1	0	0	1	2
0.00	0	0	0	1	0	0	1	2
1.00	1	0	0	0	0	0	1	1
0.00	0	0	0	1	0	0	1	2
0.00	0	0	0	1	0	0	1	2
1.00	0	0	0	1	0	0	1	2
1.00	1	0	0	0	0	0	0	1
0.00	0	0	0	0	1	1	0	2
1.00	0	0	1	0	0	0	1	2
1.00	1	0	0	0	0	0	0	1
1.00	0	0	1	0	0	0	1	2
1.00	0	0	1	0	0	0	1	2
1.00	0	1	0	0	0	0	1	1
1.00	1	0	0	0	0	0	1	1
1.00	0	0	1	0	0	0	0	2
1.00	0	0	0	1	0	0	1	2
0.00	0	0	1	0	0	0	1	2
1.00	0	1	0	0	0	1	0	1
1.00	0	0	0	0	1	0	1	2
1.00	0	0	0	1	0	0	1	2
1.00	0	0	0	1	0	0	1	2
0.00	0	0	0	0	1	0	1	2
1.00	0	0	0	0	0	1	0	1
0.00	0	1	0	0	0	0	1	1
0.00	0	0	0	1	0	1	0	2
0.00	0	0	0	1	0	0	0	2
0.00	0	1	0	0	0	0	1	1
0.00	0	0	0	0	0	0	0	1
0.00	0	0	0	0	1	0	1	2
0.00	0	0	0	0	1	0	1	2
1.00	0	0	0	0	1	0	1	2
0.00	0	0	0	1	0	1	0	2
1.00	0	0	0	0	1	0	0	2
0.00	0	0	0	1	0	0	0	2
0.00	1	0	0	0	0	0	0	1
0.00	0	0	0	0	1	0	1	2
1.00	0	1	0	0	0	0	0	1
0.00	1	0	0	0	0	0	1	1
0.00	0	1	0	0	0	0	1	1
0.00	0	0	1	0	0	0	1	2
0.00	0	0	0	0	1	0	1	2
0.00	0	0	0	0	1	0	1	2
1.00	1	0	0	0	0	0	1	1
1.00	0	0	0	1	0	0	1	2
1.00	0	0	0	0	1	0	1	2
1.00	0	0	0	0	1	0	1	2
0.00	0	0	1	0	0	0	1	2
0.00	0	1	0	0	0	0	1	1
0.00	0	0	0	0	1	1	0	2
1.00	0	0	0	0	1	0	1	2

0.00	1	0	0	0	0	1	0	1
1.00	1	0	0	0	0	0	1	1
1.00	1	0	0	0	0	0	0	1
0.00	1	0	0	0	0	1	0	1
1.00	0	0	0	0	1	0	1	2
1.00	0	0	1	0	0	0	1	2
0.00	1	0	0	0	0	0	0	1
0.00	1	0	0	0	0	1	0	1
0.00	0	0	0	1	0	0	1	2
0.00	0	0	1	0	0	0	1	2
0.00	0	0	0	1	0	0	1	2
0.00	0	0	0	1	0	1	0	2
1.00	1	0	0	0	0	0	0	1
1.00	0	0	0	1	0	0	1	2
1.00	0	1	0	0	0	1	0	1
0.00	0	0	0	0	1	0	1	2
0.00	1	0	0	0	0	1	0	1
0.00	0	1	0	0	0	0	1	1
0.00	0	0	1	0	0	0	0	2
1.00	0	0	0	0	1	1	0	2
0.00	0	0	0	1	0	0	1	2
0.00	0	0	0	0	1	0	1	2
0.00	0	0	0	1	0	0	0	2
1.00	0	0	0	0	1	0	0	2
0.00	1	0	0	0	0	0	0	1
0.00	0	0	1	0	0	0	1	2
0.00	0	0	0	1	0	1	0	2
0.00	0	0	0	0	1	0	1	2
0.00	0	1	0	0	0	0	0	1
0.00	0	0	0	0	0	0	0	1
0.00	1	0	0	0	0	1	0	1
1.00	1	0	0	0	0	0	0	1
0.00	0	1	0	0	0	0	0	1
1.00	0	0	0	0	0	0	0	1
0.00	0	0	0	0	0	0	0	1
0.00	0	0	0	0	0	0	0	1
0.00	1	0	0	0	0	0	0	1
0.00	1	0	0	0	0	0	0	1
1.00	0	0	0	0	1	0	0	2
1.00	1	0	0	0	0	1	0	1
1.00	1	0	0	0	0	1	0	1
0.00	0	0	0	0	1	0	1	2
1.00	0	0	0	0	0	0	0	1
1.00	1	0	0	0	0	0	0	1
1.00	0	0	0	0	0	0	0	1
1.00	0	0	0	0	0	1	0	1
1.00	0	0	1	0	0	0	0	2
0.00	0	0	0	0	0	0	0	1
1.00	1	0	0	0	0	0	0	1
1.00	0	1	0	0	0	0	0	1

0.00	1	0	0	0	0	0	0	1
0.00	1	0	0	0	0	0	0	1
0.00	1	0	0	0	0	0	0	1
0.00	1	0	0	0	0	0	0	1
0.00	0	0	0	0	0	0	0	1
1.00	0	0	1	0	0	0	0	2
1.00	0	1	0	0	0	1	0	1
0.00	1	0	0	0	0	0	0	1
1.00	0	1	0	0	0	0	0	1
0.00	0	0	0	0	0	0	0	1
0.00	0	0	1	0	0	1	0	2
0.00	1	0	0	0	0	1	0	1
0.00	1	0	0	0	0	0	0	1
0.00	0	0	0	1	0	1	0	2
0.00	1	0	0	0	0	0	1	1
0.00	0	0	1	0	0	1	0	2
0.00	0	1	0	0	0	0	0	1
0.00	1	0	0	0	0	0	0	1
0.00	1	0	0	0	0	1	0	1
0.00	1	0	0	0	0	0	0	1
1.00	1	0	0	0	0	0	0	1
1.00	1	0	0	0	0	0	0	1
1.00	1	0	0	0	0	0	0	1
1.00	1	0	0	0	0	1	0	1
0.00	0	1	0	0	0	0	0	1
1.00	1	0	0	0	0	0	0	1
1.00	1	0	0	0	0	0	0	1
1.00	1	0	0	0	0	0	0	1
0.00	1	0	0	0	0	0	0	1
1.00	1	0	0	0	0	0	0	1
0.00	0	0	0	0	0	0	0	1
0.00	0	1	0	0	0	0	0	1
0.00	1	0	0	0	0	0	0	1
1.00	0	1	0	0	0	0	0	1
0.00	0	0	0	0	1	0	0	2
0.00	0	0	0	0	1	0	0	2
0.00	0	0	0	0	0	0	0	1
0.00	0	1	0	0	0	0	0	1
1.00	0	0	0	1	0	0	0	2
1.00	0	1	0	0	0	1	0	1
1.00	0	0	1	0	0	0	0	2
0.00	0	0	0	0	0	0	0	1
0.00	0	0	1	0	0	0	0	2
0.00	1	0	0	0	0	0	0	1
0.00	1	0	0	0	0	0	0	1
0.00	0	0	0	0	0	0	0	1
0.00	0	0	0	0	0	0	0	1
0.00	0	0	0	0	0	0	0	1
0.00	0	0	0	0	0	0	0	1
0.00	1	0	0	0	0	1	0	1

0.00	0	0	0	0	1	0	0	2
0.00	0	0	1	0	0	1	0	2
0.00	0	0	0	0	1	1	0	2

income_r	OHL_level	MNA_level	IADL_level	MNA_2leve	OHL_2leve	behavior_l	behavior_3	behavior_n
1	1	3	2	2	1	2.00	2.00	2.00
1	1	3	1	2	1	2.00	2.00	2.00
1	2	3	2	2	2	2.00	3.00	1.00
2	3	2	2	1	2	2.00	2.00	2.00
2	2	3	2	2	1	2.00	3.00	1.00
1	2	3	2	2	1	2.00	3.00	1.00
1	2	3	1	2	2	1.00	1.00	1.00
2	3	3	2	2	2	2.00	2.00	2.00
2	3	3	2	2	2	2.00	3.00	1.00
2	3	3	2	1	2	2.00	3.00	1.00
2	3	3	2	1	2	1.00	2.00	2.00
2	3	3	2	2	2	2.00	2.00	2.00
2	3	2	1	1	2	1.00	2.00	2.00
1	3	3	1	1	2	1.00	2.00	2.00
1	1	3	2	2	1	2.00	2.00	2.00
1	3	3	1	2	2	1.00	2.00	2.00
1	3	3	2	2	2	1.00	2.00	2.00
1	3	3	2	1	2	2.00	3.00	1.00
1	1	2	2	1	1	1.00	2.00	2.00
2	1	3	2	1	1	2.00	2.00	2.00
2	3	3	2	1	2	1.00	2.00	2.00
2	3	3	1	2	2	1.00	1.00	1.00
2	3	3	2	2	2	2.00	3.00	1.00
2	3	3	2	2	2	2.00	3.00	1.00
1	1	3	1	2	1	2.00	2.00	2.00
1	2	2	1	1	1	2.00	2.00	2.00
1	1	1	1	1	1	1.00	2.00	2.00
2	3	3	2	2	2	2.00	2.00	2.00
2	2	3	1	2	2	1.00	2.00	2.00
2	3	3	2	2	2	2.00	2.00	2.00
2	3	3	2	2	2	2.00	3.00	1.00
2	3	3	2	2	2	2.00	2.00	2.00
2	2	3	2	2	2	2.00	2.00	2.00
2	1	3	1	1	1	2.00	2.00	2.00
2	2	3	1	1	2	1.00	2.00	2.00
2	2	3	1	2	1	1.00	1.00	1.00
2	2	2	2	1	1	2.00	3.00	1.00
2	1	3	2	1	1	2.00	2.00	2.00
2	1	3	1	2	1	2.00	2.00	2.00
2	3	3	2	2	2	1.00	2.00	2.00
1	1	3	1	2	1	2.00	3.00	1.00
2	2	3	2	1	2	2.00	3.00	1.00
2	2	3	1	1	2	1.00	2.00	2.00
1	3	3	2	2	2	2.00	2.00	2.00
2	3	3	2	2	2	2.00	2.00	2.00
2	2	3	1	2	1	2.00	3.00	1.00
2	1	3	1	2	1	1.00	2.00	2.00
1	2	3	1	2	2	2.00	3.00	1.00
2	1	2	1	1	1	1.00	2.00	2.00

2	3	3	2	1	2	2.00	2.00	2.00
2	3	3	2	2	2	2.00	2.00	2.00
2	2	3	1	2	2	2.00	2.00	2.00
2	3	3	2	2	2	2.00	2.00	2.00
2	3	3	2	2	2	2.00	2.00	2.00
2	3	3	2	2	2	1.00	2.00	2.00
1	1	3	1	2	1	2.00	2.00	2.00
2	2	3	1	2	2	2.00	2.00	2.00
2	3	3	1	2	2	2.00	2.00	2.00
1	3	3	2	2	2	1.00	2.00	2.00
2	1	3	2	2	1	2.00	3.00	1.00
2	3	3	1	1	2	2.00	2.00	2.00
2	3	3	1	1	2	1.00	2.00	2.00
2	1	2	2	1	1	1.00	2.00	2.00
1	3	3	1	1	2	1.00	1.00	1.00
2	3	2	1	1	2	1.00	1.00	1.00
2	3	3	1	1	2	1.00	2.00	2.00
2	1	2	2	1	1	2.00	2.00	2.00
2	3	3	1	2	2	2.00	2.00	2.00
2	3	3	2	2	2	2.00	2.00	2.00
2	3	3	2	2	2	2.00	2.00	2.00
2	3	3	2	2	2	2.00	2.00	2.00
2	1	2	1	1	1	1.00	2.00	2.00
2	1	3	2	2	1	2.00	3.00	1.00
2	3	3	2	1	2	1.00	1.00	1.00
1	1	3	2	1	1	1.00	1.00	1.00
2	2	3	2	2	1	1.00	1.00	1.00
1	1	2	1	1	1	2.00	2.00	2.00
2	2	3	2	2	2	2.00	3.00	1.00
2	3	2	2	1	2	2.00	2.00	2.00
2	3	3	2	2	2	2.00	2.00	2.00
2	3	2	1	1	2	1.00	2.00	2.00
1	1	3	2	2	1	2.00	2.00	2.00
1	2	3	2	2	2	2.00	2.00	2.00
1	1	3	2	2	1	1.00	2.00	2.00
2	3	3	2	2	2	2.00	2.00	2.00
1	1	3	2	1	1	2.00	2.00	2.00
2	1	2	2	1	1	1.00	2.00	2.00
2	1	3	2	2	1	2.00	2.00	2.00
2	1	3	2	1	1	1.00	2.00	2.00
2	1	3	2	2	1	1.00	1.00	1.00
2	3	3	2	1	2	1.00	2.00	2.00
2	1	3	2	2	1	2.00	3.00	1.00
2	3	3	1	2	2	1.00	2.00	2.00
2	3	2	1	1	2	1.00	2.00	2.00
2	1	3	2	2	1	1.00	1.00	1.00
2	3	3	2	1	2	1.00	2.00	2.00
2	1	3	2	1	1	1.00	2.00	2.00
2	2	1	1	1	2	1.00	2.00	2.00
2	1	3	2	1	1	1.00	1.00	1.00

2	2	3	1	2	1	2.00	2.00	2.00
2	3	3	1	2	2	2.00	2.00	2.00
1	3	3	2	2	2	2.00	3.00	1.00
2	1	3	2	2	1	1.00	2.00	2.00
2	3	3	1	2	2	2.00	2.00	2.00
2	3	3	2	2	2	1.00	2.00	2.00
1	1	3	2	2	1	2.00	2.00	2.00
2	2	3	2	2	1	2.00	2.00	2.00
2	3	3	1	2	2	2.00	2.00	2.00
2	1	3	2	2	1	2.00	2.00	2.00
2	3	3	2	2	2	2.00	2.00	2.00
2	3	3	1	2	2	2.00	2.00	2.00
1	1	3	1	2	1	1.00	2.00	2.00
2	3	3	2	2	2	2.00	2.00	2.00
2	3	3	1	2	2	1.00	1.00	1.00
2	1	3	2	2	1	1.00	2.00	2.00
2	1	2	2	1	1	1.00	2.00	2.00
2	1	3	2	2	1	1.00	2.00	2.00
1	1	3	2	1	1	1.00	1.00	1.00
2	3	3	2	2	2	2.00	2.00	2.00
2	1	2	2	1	1	2.00	2.00	2.00
2	1	3	2	2	1	2.00	2.00	2.00
1	1	3	2	2	1	1.00	2.00	2.00
1	1	3	2	2	1	1.00	2.00	2.00
1	1	3	2	1	1	1.00	1.00	1.00
2	3	3	2	2	2	1.00	1.00	1.00
2	2	3	2	2	1	1.00	1.00	1.00
2	1	3	2	1	1	1.00	2.00	2.00
1	3	2	1	1	2	1.00	1.00	1.00
1	1	1	1	1	1	1.00	2.00	2.00
2	1	1	1	1	1	2.00	2.00	2.00
1	2	1	1	1	1	2.00	2.00	2.00
1	2	3	2	2	2	2.00	2.00	2.00
1	1	1	1	1	1	1.00	2.00	2.00
1	1	3	1	2	1	1.00	2.00	2.00
1	1	3	1	2	1	1.00	2.00	2.00
1	2	3	1	2	2	1.00	2.00	2.00
1	1	3	1	2	1	1.00	2.00	2.00
1	1	1	1	1	1	1.00	2.00	2.00
2	3	3	2	2	2	2.00	2.00	2.00
2	2	3	2	2	2	2.00	2.00	2.00
2	3	3	2	2	2	1.00	1.00	1.00
1	1	3	1	2	1	2.00	2.00	2.00
1	1	1	1	1	1	1.00	1.00	1.00
1	1	2	1	1	1	2.00	2.00	2.00
2	1	1	1	1	1	1.00	2.00	2.00
1	3	2	1	1	2	2.00	2.00	2.00
1	1	2	1	1	1	2.00	2.00	2.00
1	1	2	1	1	1	1.00	2.00	2.00
1	1	2	1	1	1	2.00	2.00	2.00

1	1	2	1	1	1	2.00	2.00	2.00
1	1	2	1	1	1	1.00	2.00	2.00
1	1	3	1	1	1	1.00	2.00	2.00
1	1	2	1	1	1	1.00	2.00	2.00
1	1	2	1	1	1	1.00	2.00	2.00
1	1	3	1	2	1	2.00	2.00	2.00
2	2	1	1	1	1	1.00	1.00	1.00
1	1	2	1	1	1	1.00	2.00	2.00
1	2	2	1	1	2	1.00	2.00	2.00
1	1	3	1	1	1	1.00	2.00	2.00
2	2	1	1	1	2	2.00	2.00	2.00
2	1	3	1	2	1	1.00	2.00	2.00
1	1	2	1	1	1	1.00	1.00	1.00
2	3	3	2	1	2	1.00	2.00	2.00
2	3	2	2	1	2	2.00	2.00	2.00
2	2	2	1	1	2	2.00	2.00	2.00
1	2	3	1	1	1	1.00	1.00	1.00
1	1	2	1	1	1	1.00	2.00	2.00
2	2	3	2	2	1	1.00	1.00	1.00
1	1	3	1	1	1	1.00	2.00	2.00
1	1	3	1	1	1	1.00	2.00	2.00
1	1	3	1	1	1	1.00	2.00	2.00
1	1	2	1	1	1	1.00	1.00	1.00
2	2	3	1	1	1	1.00	2.00	2.00
1	1	3	1	2	1	1.00	1.00	1.00
1	1	2	1	1	1	1.00	2.00	2.00
1	1	2	1	1	1	1.00	1.00	1.00
1	1	2	1	1	1	1.00	1.00	1.00
1	1	3	1	2	1	1.00	1.00	1.00
1	1	1	1	1	1	1.00	1.00	1.00
1	1	2	1	1	1	1.00	1.00	1.00
1	1	1	1	1	1	1.00	1.00	1.00
1	1	2	1	1	1	2.00	2.00	2.00
1	2	2	1	1	1	2.00	2.00	2.00
1	2	3	1	1	1	1.00	1.00	1.00
1	2	2	1	1	2	1.00	2.00	2.00
1	1	3	1	2	1	2.00	2.00	2.00
1	2	3	1	2	1	2.00	2.00	2.00
1	3	2	1	1	2	1.00	2.00	2.00
2	2	2	1	1	2	2.00	2.00	2.00
1	3	2	1	1	2	2.00	2.00	2.00
1	1	2	1	1	1	2.00	2.00	2.00
1	1	2	1	1	1	2.00	2.00	2.00
1	1	2	1	1	1	1.00	2.00	2.00
1	2	3	1	2	2	2.00	2.00	2.00
1	1	1	1	1	1	1.00	2.00	2.00
1	1	3	1	1	1	2.00	2.00	2.00
1	1	3	1	1	1	2.00	2.00	2.00
1	1	2	1	1	1	1.00	2.00	2.00
2	2	2	1	1	1	1.00	2.00	2.00

1	3	2	1	1	2	2.00	2.00	2.00
2	2	2	1	1	2	2.00	2.00	2.00
2	3	3	1	2	2	2.00	2.00	2.00

quality_r1	quality_r2	quality_r3	quality_r4	quality_r5	quality_r6	quality_r7	quality_r8	quality_r9
3	3	3	2	2	2	2	2	2
2	2	2	2	2	2	2	2	3
4	4	2	4	3	4	4	4	4
4	3	4	2	2	3	3	4	4
4	4	4	4	3	4	4	4	4
4	4	4	4	4	4	4	4	4
2	4	3	4	4	4	4	4	4
4	4	4	4	4	4	4	4	4
4	3	3	3	3	3	4	2	4
4	4	4	4	4	4	4	4	4
4	4	4	2	2	2	4	3	4
4	4	3	4	4	4	4	4	4
4	4	4	4	4	4	4	4	4
1	2	3	4	4	4	4	3	3
4	4	4	4	4	4	4	4	4
3	4	4	4	3	4	4	4	4
4	4	4	4	4	4	4	4	4
4	4	4	4	4	4	4	4	4
3	2	2	2	2	4	3	3	4
3	3	3	3	3	3	3	3	3
3	4	4	4	3	4	4	4	4
4	4	2	2	1	2	4	4	4
4	4	4	4	4	4	4	4	4
4	4	4	4	2	3	4	3	3
4	4	4	4	4	4	3	4	4
4	4	4	4	4	4	4	4	4
3	3	3	2	3	3	3	3	3
3	4	2	2	2	2	2	4	3
4	4	4	2	2	1	2	3	2
4	4	4	2	3	2	3	3	3
4	4	2	2	2	2	3	4	2
2	3	2	1	1	1	1	3	2
3	4	4	3	3	4	4	4	4
3	3	4	3	3	3	3	3	3
3	4	4	3	3	3	4	4	3
2	2	2	1	2	2	2	2	3
3	4	3	3	2	3	4	4	4
2	4	3	2	3	3	3	4	3
2	2	2	2	3	3	3	2	2
4	4	4	4	4	4	4	4	4
2	3	4	4	4	4	3	2	2
4	4	4	4	4	4	4	4	4
3	4	4	4	2	3	2	4	3
4	4	4	2	2	2	1	4	4
4	4	3	4	3	4	4	4	4
4	4	4	4	4	4	4	4	4
2	3	2	3	2	2	2	3	2
3	3	2	3	2	2	2	3	2
3	3	2	3	3	3	2	3	3

1	1	2	2	2	2	2	3	1
4	4	4	4	4	4	3	4	4
2	2	3	2	3	3	2	3	3
4	4	4	4	4	4	4	4	4
4	4	4	4	3	3	4	4	4
3	3	3	2	2	2	2	3	2
3	4	4	3	4	4	2	4	3
4	4	4	4	4	4	4	4	4
2	3	2	2	2	2	3	3	2
2	3	3	2	2	2	2	3	3
3	3	2	2	3	3	2	4	3
4	4	4	3	3	4	2	4	3
3	3	3	2	3	3	3	4	3
2	3	2	3	2	3	3	3	3
4	3	3	3	3	2	3	3	3
3	3	4	2	2	3	2	3	3
3	4	4	4	2	3	4	4	4
2	3	4	4	4	1	4	4	4
3	4	1	2	2	2	3	4	2
2	3	2	3	3	2	2	2	3
3	3	3	3	3	3	3	3	3
4	4	4	4	4	4	4	4	4
4	3	4	3	2	4	3	3	4
4	2	4	4	2	4	4	4	4
4	3	4	4	4	4	3	4	4
4	4	4	4	4	4	4	4	4
4	4	4	4	3	3	4	4	4
4	4	4	4	4	4	4	4	4
4	4	4	4	4	4	4	4	4
4	4	4	4	4	4	4	4	4
4	4	4	2	2	2	4	4	3
4	4	4	4	3	4	4	4	4
3	3	3	4	3	2	3	4	4
4	4	4	4	4	3	4	4	4
4	4	4	4	4	4	4	3	4
4	4	4	4	4	4	4	4	4
2	2	2	2	2	2	3	4	2
4	4	4	4	3	4	4	4	4
4	4	4	4	2	3	4	4	4
4	4	4	4	4	4	4	4	4
3	4	4	4	4	3	4	4	4
4	4	4	4	4	4	4	4	4
3	3	3	2	3	3	3	4	3
3	3	4	2	2	2	2	2	1
2	1	4	1	1	2	1	2	2
4	4	4	3	1	1	2	3	3
4	4	4	3	3	3	4	4	4
3	3	4	4	4	4	3	4	4
4	4	4	4	4	4	4	4	4
2	4	3	3	3	3	3	4	4

4	4	4	4	4	4	4	4	4
4	4	2	2	2	3	3	3	3
2	4	4	3	3	3	2	4	4
3	4	4	4	4	4	2	3	4
4	4	2	2	1	1	3	4	3
4	4	4	3	3	2	3	3	3
3	3	4	4	4	4	4	4	4
4	4	4	3	3	3	4	4	4
4	4	4	2	3	3	4	4	4
4	4	4	4	4	4	4	4	4
4	4	4	4	4	4	4	4	4
4	4	4	4	4	4	4	4	4
3	3	3	3	3	3	2	3	3
4	4	4	2	2	3	2	2	2
3	3	3	3	3	3	3	3	3
4	4	4	2	4	4	4	3	4
4	4	4	4	4	3	4	4	4
4	4	4	4	3	3	4	3	4
4	4	3	4	4	3	3	4	4
2	4	4	4	1	3	4	3	4
4	4	4	4	4	4	4	4	4
4	4	4	4	3	3	4	4	4
4	4	4	4	2	2	4	4	4
4	4	4	4	2	1	4	4	3
4	4	4	3	4	4	4	4	4
4	2	4	3	4	3	4	4	4
4	4	4	3	3	4	4	4	3
4	4	4	4	4	3	4	4	4
4	2	4	4	4	4	4	4	4
4	3	4	3	4	4	3	4	4
4	4	4	4	4	4	4	4	4
3	3	3	3	3	3	3	3	3
4	4	4	4	4	4	4	4	4
3	3	3	3	3	3	3	3	3
4	4	4	4	4	4	4	4	4
4	4	4	4	4	4	4	4	4
4	4	4	4	4	4	4	4	4
4	4	4	4	4	4	4	4	4
3	3	3	3	3	3	3	3	3
4	4	4	4	4	3	3	2	3
3	4	3	3	3	2	3	3	3
4	4	4	4	4	4	4	4	4
4	3	2	3	2	2	3	3	3
2	2	2	2	2	3	2	2	2
3	3	3	3	3	3	3	3	3
2	2	2	2	2	2	2	3	3
3	3	3	3	3	3	3	3	3
4	4	4	4	4	4	4	4	4
3	3	3	3	3	3	3	3	3
3	3	3	3	3	3	3	3	3

4	4	4	4	4	4	4	4	4
4	4	4	4	4	4	4	4	4
3	3	4	3	4	4	4	4	4
4	4	4	4	4	4	4	4	4
4	4	4	4	4	4	4	4	4
3	3	3	3	3	3	3	3	3
4	2	3	2	2	2	2	3	3
3	4	4	4	4	4	4	4	4
3	3	3	3	3	3	3	3	3
3	3	4	4	4	4	4	4	4
4	4	4	4	4	4	4	4	4
4	4	4	4	4	4	4	4	4
4	4	4	4	4	4	4	4	4
4	4	4	4	4	4	4	4	4
4	4	4	4	4	4	4	4	4
4	4	4	4	4	4	4	4	4
4	4	4	4	4	4	4	4	4
4	4	4	4	4	4	4	4	4
4	4	4	4	4	4	4	4	4
4	4	4	4	4	4	4	4	4
3	3	3	3	3	3	3	3	3
3	3	3	3	3	3	3	3	3
3	2	3	3	3	3	3	3	3
3	3	3	3	3	3	3	3	3
4	4	4	4	4	4	4	4	4
3	3	3	3	3	3	3	3	3
3	3	3	3	3	3	3	3	3
3	3	3	3	3	3	3	3	3
4	4	4	4	4	4	4	4	4
3	3	3	3	3	3	3	3	3
4	4	4	4	4	4	4	4	4
4	4	4	4	4	4	4	4	4
4	4	4	4	4	4	4	4	4
3	3	3	2	3	3	3	3	4
4	4	4	4	4	4	4	4	4
4	4	4	4	4	4	4	4	4
4	4	4	4	4	4	4	4	4
4	4	4	4	4	4	4	4	4
3	3	3	3	3	3	3	3	3
3	3	3	3	3	3	2	4	4
3	3	3	3	3	3	3	3	3
4	4	4	4	4	4	4	4	4
4	4	4	4	4	4	4	4	4
4	4	4	4	4	4	4	4	4
4	4	4	4	4	4	4	4	4
4	4	4	4	4	4	4	4	4
4	4	4	4	4	4	4	4	4
4	4	4	4	4	4	4	4	4
4	4	4	4	4	4	4	4	4
4	4	4	4	4	4	4	4	4

4	4	4	4	4	4	4	4	4
4	4	4	4	4	4	4	4	4
4	4	4	4	4	4	4	4	4

quality_r10	quality_r11	quality_r12	quality_r13	quality_r14	quality_rto	quality_rpoor
2	2	2	2	2	31	0
2	3	4	3	4	35	0
4	4	4	4	4	53	0
4	4	4	3	4	48	0
4	4	4	4	4	55	0
4	4	4	4	4	56	1
4	4	4	4	4	53	0
4	4	4	4	4	56	1
4	4	4	4	4	49	0
4	4	4	4	4	56	1
4	4	3	3	3	46	0
4	4	4	3	4	54	0
4	4	4	4	4	56	1
3	3	3	3	3	43	0
4	4	4	4	4	56	1
4	4	4	4	4	54	0
4	4	4	4	4	56	1
4	4	4	4	4	56	1
4	4	4	2	4	43	0
3	3	3	3	3	42	0
4	4	4	4	4	54	0
4	4	4	3	4	46	0
4	4	4	4	4	56	1
4	4	4	4	4	51	0
4	4	4	3	4	54	0
4	4	4	4	4	56	1
3	3	3	3	3	41	0
3	3	4	3	3	40	0
2	2	2	3	4	37	0
4	3	4	3	3	45	0
2	3	4	4	4	42	0
1	2	2	2	2	25	0
3	4	4	4	4	52	0
3	3	4	4	4	46	0
4	3	3	3	4	48	0
1	2	3	2	2	28	0
4	4	4	3	4	49	0
2	3	3	3	3	41	0
2	3	3	1	2	32	0
4	4	4	4	4	56	1
3	2	3	2	3	41	0
4	4	4	4	4	56	1
3	4	4	2	4	46	0
1	4	4	3	4	43	0
4	4	4	4	4	54	0
2	4	4	2	2	50	0
4	3	3	4	4	39	0
3	2	3	2	3	35	0
4	3	3	2	3	40	0

3	3	2	2	3	29	0
4	4	4	3	4	54	0
3	3	3	2	3	37	0
4	4	4	4	4	56	1
4	4	4	4	4	54	0
2	4	4	2	4	38	0
4	3	4	4	4	50	0
4	4	4	4	4	56	1
3	3	3	3	3	36	0
3	2	3	3	3	36	0
4	3	3	3	3	41	0
3	4	4	4	4	50	0
4	4	4	4	4	47	0
3	3	3	2	3	38	0
2	4	3	3	3	42	0
2	2	4	2	4	39	0
3	4	4	4	4	51	0
4	4	4	4	2	48	0
3	4	3	3	3	39	0
2	3	3	2	2	34	0
3	3	3	3	3	42	0
4	4	4	4	4	56	1
4	4	4	4	4	50	0
4	4	4	4	4	52	0
4	4	4	4	4	54	0
3	4	4	4	4	55	0
4	4	4	4	4	54	0
4	4	4	4	4	56	1
4	4	4	4	4	56	1
4	4	4	4	4	56	1
4	4	4	3	4	48	0
4	4	4	4	4	55	0
3	3	3	3	4	45	0
4	4	4	4	4	55	0
4	4	4	3	4	54	0
4	4	4	4	4	56	1
2	2	4	3	3	35	0
4	4	4	4	4	55	0
4	4	4	4	4	53	0
4	4	4	4	4	56	1
4	4	3	4	3	52	0
4	4	4	4	4	56	1
3	4	4	4	4	46	0
2	2	3	2	3	33	0
3	3	3	2	3	30	0
1	4	4	1	3	38	0
4	4	4	4	4	53	0
4	4	4	3	3	51	0
4	4	4	4	4	56	1
4	4	4	4	4	49	0

4	4	4	4	4	56	1
2	3	3	4	3	41	0
3	3	3	4	3	45	0
4	4	4	4	4	52	0
4	4	4	3	4	43	0
4	3	3	3	3	45	0
4	4	4	4	4	54	0
4	4	4	4	4	53	0
4	4	4	4	4	52	0
4	4	4	4	4	56	1
4	4	4	4	4	56	1
4	4	4	4	4	56	1
3	3	3	3	3	41	0
3	3	4	2	3	40	0
3	3	3	3	3	42	0
4	4	4	4	4	53	0
4	4	4	4	4	55	0
4	4	4	4	4	53	0
4	4	4	4	4	53	0
4	4	4	4	4	49	0
4	3	4	4	2	53	0
4	4	4	4	4	54	0
3	4	4	4	4	51	0
4	4	4	4	4	50	0
4	4	4	4	4	55	0
4	4	4	4	4	52	0
4	4	4	4	4	53	0
3	4	4	4	4	54	0
4	4	4	4	4	54	0
4	4	4	3	4	52	0
4	4	4	4	4	56	1
3	3	3	3	3	42	0
4	4	4	4	4	56	1
3	3	3	3	3	42	0
4	4	4	4	4	56	1
4	4	4	4	4	56	1
4	4	4	4	4	56	1
4	4	4	4	4	56	1
3	3	3	3	3	42	0
4	4	4	4	3	50	0
4	4	4	3	3	45	0
4	4	4	4	4	56	1
3	3	3	3	3	40	0
2	2	2	2	2	29	0
3	3	3	3	3	42	0
3	3	3	3	3	35	0
3	3	3	3	3	42	0
4	4	4	4	4	56	1
3	3	3	3	3	42	0
3	3	3	3	3	42	0

4	4	4	4	4	56	1
4	4	4	4	4	56	1
4	4	4	4	4	53	0
4	4	4	4	4	56	1
4	4	4	4	4	56	1
3	3	3	3	3	42	0
3	3	2	2	2	35	0
4	4	4	4	4	55	0
3	3	4	3	3	43	0
4	4	4	4	4	54	0
4	4	4	4	4	56	1
4	4	4	4	4	56	1
4	4	4	4	4	56	1
4	4	4	4	4	56	1
4	4	4	4	4	56	1
4	4	4	4	4	56	1
4	4	4	4	4	56	1
4	4	4	4	4	56	1
4	4	4	4	4	56	1
4	4	4	4	4	56	1
3	3	3	3	3	42	0
3	3	3	3	3	42	0
3	3	3	3	3	41	0
4	4	4	4	4	47	0
4	4	4	4	4	56	1
3	3	3	3	3	42	0
3	3	3	3	3	42	0
3	3	3	3	3	42	0
4	4	4	4	4	56	1
3	3	3	3	3	42	0
4	4	4	4	4	56	1
4	4	4	4	4	56	1
4	4	4	4	4	56	1
4	4	4	3	3	45	0
4	4	4	4	4	56	1
4	4	4	4	4	56	1
4	4	4	4	4	56	1
4	4	4	4	4	56	1
3	4	4	3	3	44	0
4	4	4	3	3	46	0
3	3	3	3	3	42	0
4	4	4	4	4	56	1
4	4	4	4	4	56	1
4	4	4	4	4	56	1
4	4	4	4	4	56	1
4	4	4	4	4	56	1
4	4	4	4	4	56	1
4	4	4	4	4	56	1
4	4	4	4	4	56	1
4	4	4	4	4	56	1

4	4	4	4	4	56	1
4	4	4	4	4	56	1
4	4	4	4	4	56	1
